# A predictive coding account of bistable perception - a model-based fMRI study

**DOI:** 10.1371/journal.pcbi.1005536

**Published:** 2017-05-15

**Authors:** Veith Weilnhammer, Heiner Stuke, Guido Hesselmann, Philipp Sterzer, Katharina Schmack

**Affiliations:** 1 Department of Psychiatry, Charité Universitätsmedizin Berlin, 10117 Berlin, Germany; 2 Bernstein Center for Computational Neuroscience, Charité Universitätsmedizin Berlin, 10117 Berlin, Germany; 3 Berlin School of Mind and Brain, Humboldt-Universität zu Berlin, 10099 Berlin, Germany; Brain and Spine Institute (ICM), FRANCE

## Abstract

In bistable vision, subjective perception wavers between two interpretations of a constant ambiguous stimulus. This dissociation between conscious perception and sensory stimulation has motivated various empirical studies on the neural correlates of bistable perception, but the neurocomputational mechanism behind endogenous perceptual transitions has remained elusive. Here, we recurred to a generic Bayesian framework of predictive coding and devised a model that casts endogenous perceptual transitions as a consequence of prediction errors emerging from residual evidence for the suppressed percept. Data simulations revealed close similarities between the model’s predictions and key temporal characteristics of perceptual bistability, indicating that the model was able to reproduce bistable perception. Fitting the predictive coding model to behavioural data from an fMRI-experiment on bistable perception, we found a correlation across participants between the model parameter encoding perceptual stabilization and the behaviourally measured frequency of perceptual transitions, corroborating that the model successfully accounted for participants’ perception. Formal model comparison with established models of bistable perception based on mutual inhibition and adaptation, noise or a combination of adaptation and noise was used for the validation of the predictive coding model against the established models. Most importantly, model-based analyses of the fMRI data revealed that prediction error time-courses derived from the predictive coding model correlated with neural signal time-courses in bilateral inferior frontal gyri and anterior insulae. Voxel-wise model selection indicated a superiority of the predictive coding model over conventional analysis approaches in explaining neural activity in these frontal areas, suggesting that frontal cortex encodes prediction errors that mediate endogenous perceptual transitions in bistable perception. Taken together, our current work provides a theoretical framework that allows for the analysis of behavioural and neural data using a predictive coding perspective on bistable perception. In this, our approach posits a crucial role of prediction error signalling for the resolution of perceptual ambiguities.

## Author summary

In bistable vision, perception spontaneously alternates between two different interpretations of a constant ambiguous stimulus. Here, we show that such spontaneous perceptual transitions can be parsimoniously described by a Bayesian predictive coding model. Using simulated, behavioural and fMRI data, we provide evidence that prediction errors stemming from the suppressed stimulus interpretation mediate perceptual transitions and correlate with neural activity in inferior frontal gyrus and insula. Our findings empirically corroborate theorizations on the relevance of prediction errors for spontaneous perceptual transitions and substantially contribute to a longstanding debate on the role of frontal activity in bistable vision. Therefore, our current work fundamentally advances our mechanistic understanding of perceptual inference in the human brain.

## Introduction

During bistable perception, observers experience fluctuations between two mutually exclusive interpretations of a constant ambiguous input. Remarkably, percepts evoked by ambiguous stimuli usually closely resemble the experience of unambiguous objects and thus illustrate the constructive nature of perception. However, the mechanisms driving transitions in bistable perception remain poorly understood.

Previous neuroimaging work [4, 5, 6, 7, 8, 9, 10] has sought to distill the neural processes underlying bistable perception by recurring to a ‘replay’ condition, in which physical stimulus changes mimic the perceptual alternations induced by ambiguous stimuli. This approach revealed a right-lateralized assembly of fronto-parietal areas whose activity is specifically enhanced during endogenously evoked transitions (ambiguity) as compared to exogenously evoked transitions (replay) [4, 5, 7, 9].

However, the functional role of fronto-parietal areas in bistable perception is a matter of ongoing debate. According to one view, transitions in bistable vision are primarily a result of adaptation and inhibition within visual cortex, while switch-related activations in fronto-parietal areas reflect a mere ‘feedforward’ consequence of neural events at sensory processing levels [6, 10]. Another view proposes that fronto-parietal areas may be involved in stabilizing and destabilizing perception, thus causally contributing to perceptual switching via ‘feedback’ mechanisms [4, 5, 11, 7]. Here, we sought to resolve this debate by using model-based fMRI to empirically test a theoretical model that has the potential to integrate these two seemingly contradictory views of perceptual bistability.

From a theoretical perspective, endogenous transitions might be explained by framing perception as an inferential process generating and testing hypotheses about the most likely causes of sensory stimulation [12, 13, 14]. Such processes can be elegantly implemented by hierarchical predictive coding [15, 16, 17]. Here, ‘predictions’ encoded at higher levels are compared against ‘sensory input’ represented at lower levels, while a mismatch between the two elicits a prediction error, updating higher-level predictions [15]. Such belief-updating schemes can be translated onto Bayes’ rule, where prior distributions (‘predictions’) are combined with likelihood distributions (’sensory input’) into posterior distributions in a sequential manner [16, 18].

Here, we tested whether this framework provides a mechanistic explanation for perceptual transitions and related neural activity during bistable perception. We devised a computational model that formalizes perceptual decisions (i.e., decisions that define the content of conscious perception, as indicated by participants’ response) to be performed on the basis of posterior probability distributions. This model is a modification of an approach introduced by [19], who propose that perceptual time-courses during bistable perception result from samples drawn subsequently from a posterior distribution. The authors implement a memory decay favoring recent over older samples as well as stationary prior capturing the effect of context on bistable perception. Our model, in turn, posits that the shape of the posterior distribution changes dynamically over time in response to prediction errors emerging from the currently suppressed interpretation of the ambiguous input. Importantly, this model has the potential to integrate feedforward and feedback mechanisms in bistable perception: The prediction errors arising from sensory processing levels may be propagated up to higher-level brain areas in a feedforward fashion. The registration of prediction errors in higher-level brain areas leads to an updating of predictions that may in turn drive perceptual switching through a feedback mechanism.

To test this hypothesis, we began with data simulations to establish that our model’s predictions match the key characteristics of perceptual bistability. We proceeded by fitting our model to behavioural data from a fMRI experiment on bistable perception [7].

In this experiment, participants viewed a Lissajous figure [42] rotating either clockwise (as viewed from above, i.e. movement of the front surface to the left) or counter-clockwise (vice versa) and indicated their current perception via button-presses. Participants were presented with alternating blocks of ambiguous and disambiguated Lissajous figures: In the ambiguous condition, we presented bistable Lissajous figures which elicited spontaneous (endogenous) alternations in perception. In the disambiguated (’replay’) condition, we mimicked the endogenous perceptual time-course by introducing exogenous perceptual switches. Ambiguous and disambiguated stimuli were constructed by presenting two Lissajous figures separately to the two eyes: In the ambiguous condition, both eyes received identical stimulation. In the replay condition, the two Lissajous figures were slightly phase-shifted against each other, biasing perception in the direction of the phase shift.

Having inverted our predictive coding approach based on behavioural data from this experiment, we investigated whether our model accurately explains individual perceptual time-courses during ambiguous and replay stimulation.

In a supplementary analysis (see S2 Text), we furthermore compared our model to three established models of bistable perception: Firstly, we tested an oscillator model [1], which is based on mutual inhibition between to competing neural populations coding for the alternative perceptual outcomes during bistable perception. Here, the currently dominant population suppresses activity in the alternative population. However, due to adaptation in the dominant population, this relation reverses over time, leading to regular oscillations in perception. Secondly, we constructed a noise-driven attractor model of bistable perception [2]. In this framework, internal and external sources of noise trigger transitions between two stable states in an attractor network, representing the two perceptual interpretations associated with a bistable stimulus. Thirdly, we tested an intermediate model [3], which contains both adaptive processes and noise. We validated our approach against these models by the use of Bayesian Model Comparison [20].

We then conducted a model-based fMRI-analysis [21] based on the predictive coding model to test whether prediction errors account for transition-related neural activity during bistability. Additionally, we compared the model-based fMRI analysis with conventional fMRI analyses using a Posterior Probability Map (PPM) approach [22].

## Methods

### Theoretical background

Our Bayesian modelling approach draws on the view that perception is an inferential process in which perceptual decisions are based on posterior distributions [13]. According to Bayes’ rule, the posterior combines information in the current sensory data (likelihood) with information from previous visual experience (prior) in a probabilistically optimal manner. Crucially, this posterior at a given moment becomes a prior for the current perceptual decision, which entails a prediction error signal that influences on the prior at the next moment. Hence, the posterior not only provides the basis for current perception, but also shapes future perception.

In line with previous theorizations [12], we reasoned that the ambiguous likelihood provides equally strong sensory evidence for two different percepts. We further hypothesized that the current percept establishes an implicit prior belief about similar percepts in the future, thereby contributing to stability of visual perception. The application of Bayes’ rules combines the likelihood for ambiguous stimuli with the stability prior into a posterior that represents stronger evidence for the dominant percept, but still contains residual evidence for the suppressed percept. While the stronger evidence for the dominant percept will again favor this percept for the upcoming perceptual decision, the residual evidence for the suppressed percept is equivalent to a prediction error that leads to an update of the stability prior.

Over time, the stability prior is weakened and the posterior shifts towards the suppressed percept, paralleled by an escalating prediction error. When the residual evidence for the suppressed percept equals the evidence for the dominant percept, the prediction error reaches a maximum and a perceptual transition is most likely to occur. Once such a transition has occurred, the process starts over again, minimizing the current prediction error.

Please note that our approach was influenced by the work of [19], who argue that bistable perception is a product of Bayesian decision making in ambiguous sensory environments. They study the effects of viewpoint context on perception of the Necker Cube and propose that bistable perception arises from sampling a bimodal posterior distribution. Here, the sample with the highest ‚weight’ determines the content of conscious perception. Key elements of their model are (1), a stationary prior, whose precision reflects interindividual differences in the effects of viewpoint context on perception of the Necker Cube and (2), a memory decay that discounts the weight associated with a sample drawn from the posterior distribution by its age and influences on the length of individual phase durations.

In contrast to [19], our model does not assume a specific memory decay process, but controls the length of phase durations by means of the dynamically updated stability prior. In analogy to the stationary viewpoint prior in [19], our model captures the influence of additional sensory evidence on perceptual decisions using a ‚stereodisparity’ distribution, whose precision determines the effectiveness of disambiguation.

**Fig 1:**
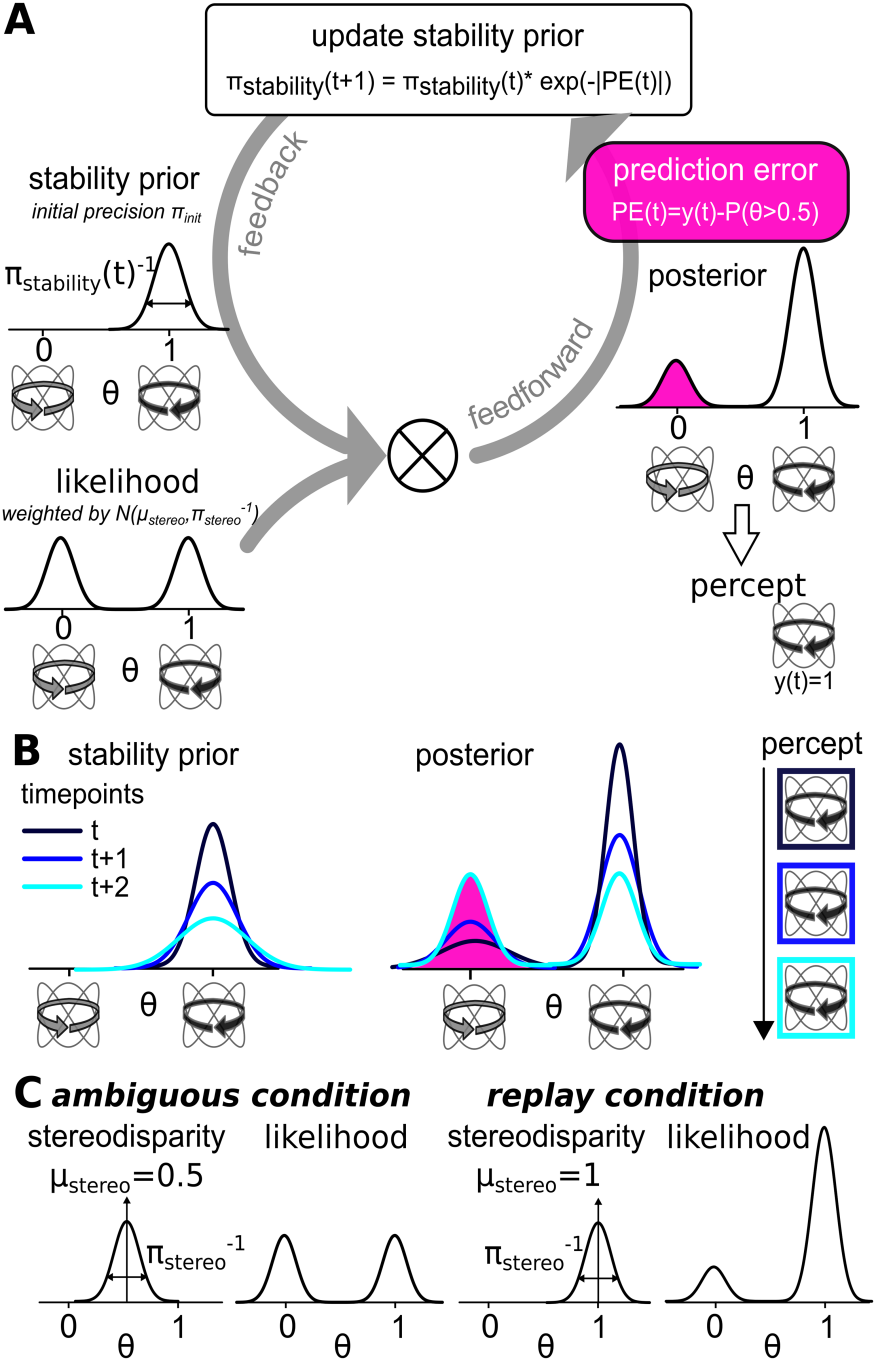
Modelling procedures. **A**. In the modelling approach illustrated here, we capture the temporal dynamics of bistable perception by changes in a continuously updated stability prior, which is combined with a bimodal likelihood representing the sensory input (see ‘feedback’ arrow). Under ambiguous viewing conditions, the likelihood contains equivalent evidence for both perceptual interpretations of the bistable stimulus. The mean of the prior ‘perceptual stability’ is defined by *μ*_*stability*_, which corresponds to the preceding perceptual decision *y*(*t* − 1) (here centered around ‘1’ for counter-clockwise rotation of the Lissajous figure). The impact of the prior on the bimodal likelihood is determined by its precision (the inverse of variance) *π*_*stability*_. If a new perceptual decision was adopted at the preceding overlapping configuration of the Lissajous figure, this precision is set to *π*_*init*_. Otherwise, *π*_*stability*_ is repeatedly updated by a prediction error signal. This signal results from residual evidence for the alternative explanation of the bistable stimulus and is given by the difference between *P*(*θ* > 0.5) and the current perceptual decision *y*(*t*) (see ‘feedforward’ arrow). In this example, the prediction error signal stems from remaining evidence for clockwise rotation (centered around ‘0’), as the current perceptual decision represents counter-clockwise rotation (*y*(*t*) = 1) of the stimulus. Overtime, the stability prior is weakened, which is accompanied by an increasing probability for a novel transition in perception. **B**. Here, we depict the temporal evolution of the stability prior (left panel) and the corresponding posterior (right panel) at three successive overlapping configurations of the Lissajous figure (dark to light blue). As the precision of the stabilizing prior is gradually reduced, the posterior relaxes to equivalent probability for both perceptual interpretations of the stimulus. This is accompanied by escalating prediction error signals and increased likelihood for a perceptual transition. **C**. Furthermore, our approach accounts for additional sensory evidence, which is realized by a stereodisparity signal and used to disambiguate the Lissajous figure in the ‘replay’ condition. To this end, we introduce a ‘stereodisparity’ distribution (characterized by mean *μ*_*stereo*_ and precision *π*_*stereo*_), which serves as a weight on the bimodal likelihood. In the ambiguous condition (left panel), *μ*_*stereo*_ is centered around 0.5 and is thus uninformative with regard to the two perceptual interpretations of the stimulus. In the replay condition (right panel), *μ*_*stereo*_ is centered around ‘0’ or ‘1’ (depending on the direction of stereodisparity). The strength of the bias in the direction of either percept introduced by the stereodisparity signal scales with the precision *π*_*stereo*_.

Please refer to to the mathematical appendix (see S1 Text) for a complete description, to Fig 1 for a step-by-step illustration of our approach and to Table 1 for a summary of model parameters and quantities. For computational expediency, we assume Gaussian probability distributions defined by mean and variance (or inverse precision).

**Table 1:** Summary of model parameters and quantities.

	Name	Explanation
**Sensory Stimulation**	*μ*_*stereo*_	Mean of sensory stimulation
**Responses**	*y*	Binary perceptual decision
**Model Parameters**	*π*_*init*_	Initial precision of stability prior
	*π*_*stereo*_	Initial precision of stability prior
	*ζ*	Inverse decision temperature of the reponse model
**Model Quantities**	*y*_*predicted*_	Predicted perceptual response
	*μ*_*stability*_	Mean of the stability prior
	*π*_*stability*_	Precision of the stability prior
	*μ*_*m*_	Mean of the joint prior
	*π*_*m*_	Precision of the joint prior
	*P*(*θ* > 0.5)	Probability of perceiving counter-clockwise rotation

### Model simulation

To test whether our model is able to reproduce the temporal dynamics of bistable perception, we used it to generate perceptual time-courses from some ambiguous visual input such as the Lissajous figure. We assumed a sampling rate of 0.33 Hz, which was chosen to be close to the average overlap frequency in the behavioural experiment (see below), and simulated for a total of 6 * 10^5^ seconds. To model the ambiguous visual input, the impact of the stereodisparity weight was suppressed by setting *μ*_*stereo*_ = 0.5 and *π*_*stereo*_ = 0. We further assumed fixed values for the precision *π*_*init*_, which was set to 3.5 to match the posterior parameter value from our behavioural modelling (see *Modelling analysis of behavioural data*).

### Experimental procedures

To examine whether our prediction error model might account for bistable perception and associated neural activity in human observers, we used data from an fMRI experiment applying the Lissajous figure. Results from conventional analyses but not from behavioural modelling or model-based fMRI (see below) have been reported previously [7].

#### Participants

Twenty right-handed participants (11 female, mean age: 28, range: 21 -34) took part in this study, which was conducted at the Berlin Center for Advanced Neuroimaging (BCAN), Charité Universitätsmedizin Berlin, Campus Mitte. All participants had normal or corrected-to-normal vision, were naive to the purpose of the study, and provided informed written consent. The study was approved by the ethics committee of Charité Universitätsmedizin Berlin, Campus Mitte.

#### Stimulus

We presented stimuli generated with Psychophysics Toolbox 3 [23] running under Matlab 2007b (Mathworks inc.) on a 60 Hz Sanyo LCD projector, on which participants viewed alternating blocks of ambiguous and corresponding replay stimulation. In ambiguous blocks, we displayed two identical moving Lissajous figures formed by the intersection of two perpendicular sinusoids (*x*(*t*) = *sin*(3*t*) and *y*(*t*) = *sin*(6*t* + *δ*); with *δ* increasing from 0 to 2*π*), separately to the two eyes. In replay blocks, a disambiguated version of the Lissajous figure mimicked the perceptual time-course participants had experienced during the preceding ambiguous block. To this end, the two dichoptically presented Lissajous figures were phase-shifted against each other by an offset of 0.04°. This disparity cue was used to disambiguate the stimulus, biasing participants perceived direction of rotation in the direction of the phase shift. All stimuli subtended 2.05° visual angle.

We achieved dichoptic stimulation by placing a custom build cardboard divider between the mirror attached to the head-coil and the screen at the end of the scanners bore [24]. Participants wore prism glasses to facilitate fusion between to two eyes. All screens contained a fixation mark at the center and fusion frames surrounding the stimuli.

#### Task

Participants were instructed to indicate the perceived direction of rotation of the Lissajous figure by pressing a left (index finger; for clockwise rotation of the stimulus, i.e.movemement of the front surface to the left) or right (ring finger) button with their right hand, responding to the first perceived direction after stimulation onset and to all additional perceptual transitions. Furthermore, they reported unclear or mixed percepts by pressing a middle button (middle finger) on a standard MRI button box.

In order to titrate individual percept durations to approximately 10 s, we adjusted the rotational speed of the stimulus for every participant to one of three levels (’overlap’ frequency 0.24, 0.30, and 0.40 Hz) based on a psychophysical experiment prior to the fMRI session. In the fMRI experiment, participants were presented with three experimental runs, each containing 8 pairs of ambiguity and replay separated by 10 s fixation. Block duration amounted to 42.8, 40.90, or 41 s, depending on the individually adjusted speed. After completion of the fMRI experiment, participants answered a debriefing questionnaire (A: *Did you have the impression that some blocks were different from others?* B: *Did you perceive the transitions as instantaneous or prolonged?* C: *Were you able to tell the direction of rotation of the Lissajous figure at all times during the experiment?*).

### fMRI acquisition and preprocessing

We recorded BOLD images by T2-weighted gradient-echo echo-planar imaging (FOV 192, 33 slices, TR 2000 ms, TE 30 ms, flip angle 78°, voxel size 3 x 3 x 3 mm, interslice gap 10 percent) on a 3T MRI scanner (Tim Trio, Siemens). The number of volumes amounted to 402 (0.15 Hz and 0.2 Hz) or 415 (0.12 Hz) volumes, respectively. We used a T1-weighted MPRAGE sequence (FOV 256, 160 slices, TR 1900 ms, TE 2.52 ms, flip angle 9°, voxel size 1 x 1 x 1 mm) to acquire anatomical images.

Image preprocessing (standard realignment, coregistration, normalization to MNI stereotactic space using unified segmentation, spatial smoothing with 8 mm full-width at half-maximum isotropic Gaussian kernel) was carried out with SPM8 (http://www.fil.ion.ucl.ac.uk/spm/software/spm8).

### Modelling analysis of behavioural data

To probe whether our predictive coding model might explain perceptual time-courses during bistable perception in human observers, we fitted our model to the behavioural data collected during the fMRI experiment. We optimized our model for the prediction of perceptual outcomes, i.e. on the perception of clockwise or counter-clockwise rotation as indicated by the individual participants. To this end, participants’ responses were aligned to the overlapping stimulus configurations of the Lissajous figure (’overlaps’). This refers to timepoints during presentation when fore- and background of the stimulus cannot be discerned (i.e. depth-symmetry) [25, 26]. Depending on the rotational speed of the stimulus and the associated ‘overlap’ frequency, sampling rates varied across participants between 0.24 Hz and 0.40 Hz (see above). We first constructed models incorporating all combinations of the likelihood weight ‘stereodisparity’ and prior ‘perceptual stability’, yielding a total of 4 behavioural models (behavioural model 1: no stereodisparity, no perceptual stability; behavioural model 2: no stereodisparity, perceptual stability; behavioural model 3: stereodisparity, no perceptual stability; behavioural model 4: stereodisparity, perceptual stability) to be compared. The respective precision of these distributions was optimized for the prediction of perceptual outcomes based on posterior distributions using a free energy minimization approach [27]. This method minimises the surprise about the individual participants’ data, thereby maximising log-model evidence.

For model inversion, precisions were modelled as log-normal distributions. *π*_*init*_ and *π*_*stereo*_ were either estimated as free parameters (*π*_*init*_: prior mean of log(3) and prior variance of 5; *π*_*stereo*_: prior mean of log(5) and prior variance of 5) or fixed to zero (thereby effectively removing the distribution from the model). We kept *ζ*, which represents the inverse decision temperature in the response model represented by Equation 11 (see Mathematical Appendix, S1 Text), fixed to 1, since we did not have a particular a-priori hypothesis regarding this parameter. Please note that when choosing *ζ* as a free parameter (prior mean of log(1), prior variance of 1), results remained almost identical. Parameters were optimised using quasi-Newton Broyden-Fletcher-Goldfarb-Shanno minimisation as implemented in the HGF4.0 toolbox (TAPAS toolbox, http://www.translationalneuromodeling.org/hgf-toolbox-v3-0/).

After identifying the optimal model using Random Effects Bayesian model selection [20], as implemented in SPM12 (http://www.fil.ion.ucl.ac.uk/spm/software/spm12/), we analyzed its posterior parameters with regard to the respective precision of the prior distributions using classical frequentist statistics. Since parameters were estimated in log-space, we report the geometric mean (i.e. the arithmetic mean in log-space).

In a supplementary analysis (see S2 Text), we further compared the explanatory power of our predictive coding model with established models of bistable perception. To this end, we implemented models of bistable perception belonging to three different classes ([1] as an example of so-called oscillator models based on mutual inhibition and self-adaptation between two competing neuronal populations, [2] as a representative of noise-driven attractor models and [3] as an intermediate model), which can be fitted to experimental data. We conducted a Random Effects Bayesian Model Comparison [20] between the established models and our predictive coding model in order to probe the validity of our approach.

### Model-based fMRI data analysis

To examine the neural correlates of prediction error time-courses from our model, we conducted model-based fMRI analyses [21] in SPM12. We adopted a general-linear-model-(GLM-) approach, constructing a total of three models:

The design matrix of the first GLM (the ‘PE model’) represented prediction error trajectories timepoint by timepoint. To this end, the regressor ‘transitions’ and the regressor ‘overlaps’ were modelled as stick functions. Furthermore, we extracted the individual ‘Prediction Error’ time-course for every participant and run and used its absolute value as a parametric modulator for the regressor ‘overlaps’.

In order to enable a comparison to the conventional approach of analysing fMRI data on bistable perception, we constructed a second GLM that dissociated between transition-related activity specific to bistable perception and the replay condition [4, 5, 6, 7, 9, 10]. In addition to the regressor ‘overlaps’, the design matrix of this ‘Conventional model’ contained ambiguous and replay transitions represented by stick functions.

To further investigate the specificity of the prediction error trajectories and their neural correlates, we constructed a third GLM that took into account the presence of ambiguity inherent to the bistable condition. The design matrix contained the regressors ‘transitions’ as well the regressor ‘overlaps’ modelled as stick functions. Here, however, we used a box-car function being 1 for ambiguous and 0 for ‘replay’ blocks as a parametric modulator of the regressor ‘overlaps’. Hence, this ‘Block model’ only differs from the ‘PE model’ in the values of the parametric modulator and serves to investigate whether correlations with the prediction error (which we assumed to be higher in the bistable condition) merely correspond to ambiguity per se.

All further analyses were conducted for all models in parallel: regressors were convolved with the canonical hemodynamic response function as implemented in SPM12. We added six rigid-body realignment parameters as nuisance covariates and applied high-pass filtering at 1/128 Hz.

In a first step, we tested which of the three models accounted best for the measured BOLD signal. Therefore, we conducted a voxel-wise model comparison of the ‘PE model’ with the ‘Conventional model’ and the ‘Block model’, as described in [22]. In brief, this technique uses Bayesian statistics for the construction of ‘Posterior Probability Maps’ (PPMs) and ‘Exceedance Probability Maps’ (EPMs), which enable the calculation of log-evidence maps for each participant and model separately. On a second level, these log-evidence maps can be combined, thereby enabling voxel-wise model inference at the group level. Using the ‘Bayesian 1st level’ procedure for model estimation, we constructed log-evidence maps for every participant and model separately and compared the ‘PE model’ to the other models on a group level using exceedance probabilities computed with Random Effects analyses.

In a second step, we aimed to identify regions in which prediction error trajectories (‘PE model’), ambiguity per se (‘Block model’) or ambiguous as compared to replay transitions (‘Conventional model’) were correlated with the recorded BOLD signals. To this end, we estimated single-participant statistical parametric maps, then created contrast images for the parametric regressor against baseline (‘PE model’ and ‘Block model’) or ambiguous against replay transitions (‘Conventional model’). These were entered into voxel-wise one-sample t-tests at the group level. Voxels were considered statistically significant if they survived family-wise-error (FWE) correction for all voxels in the brain at *p* < 0.05. Anatomic labeling of cluster peaks was performed using the SPM Anatomy Toolbox Version 1.7b [28].

In order to further visualize our results, we extracted eigenvariate time-courses (without adjustment for effects of interest) from spherical ROIs (radius: 3 mm) around peak voxels from clusters for the contrast ‘Prediction Error vs baseline’ (thresholded at *p* < 0.05) corresponding to left IFG (peak voxel: [-54 2 22]), right IFG (peak voxel: [51 8 10]), left insula (peak voxel: [-30 20 10]) and right insula (peak voxel: [33 23 7]). These time-courses were extracted for ambiguous stimulation only. The time-courses for all perceptual phases were aligned with the respect to the end of the perceptual phase and averaged within and across observers.

## Results

### Model simulation

To test whether our predictive coding model was able to reproduce perceptual switching in bistable perception, we used the model to generate perceptual time-courses during simulated viewing of an ambiguous stimulus.

**Fig 2:**
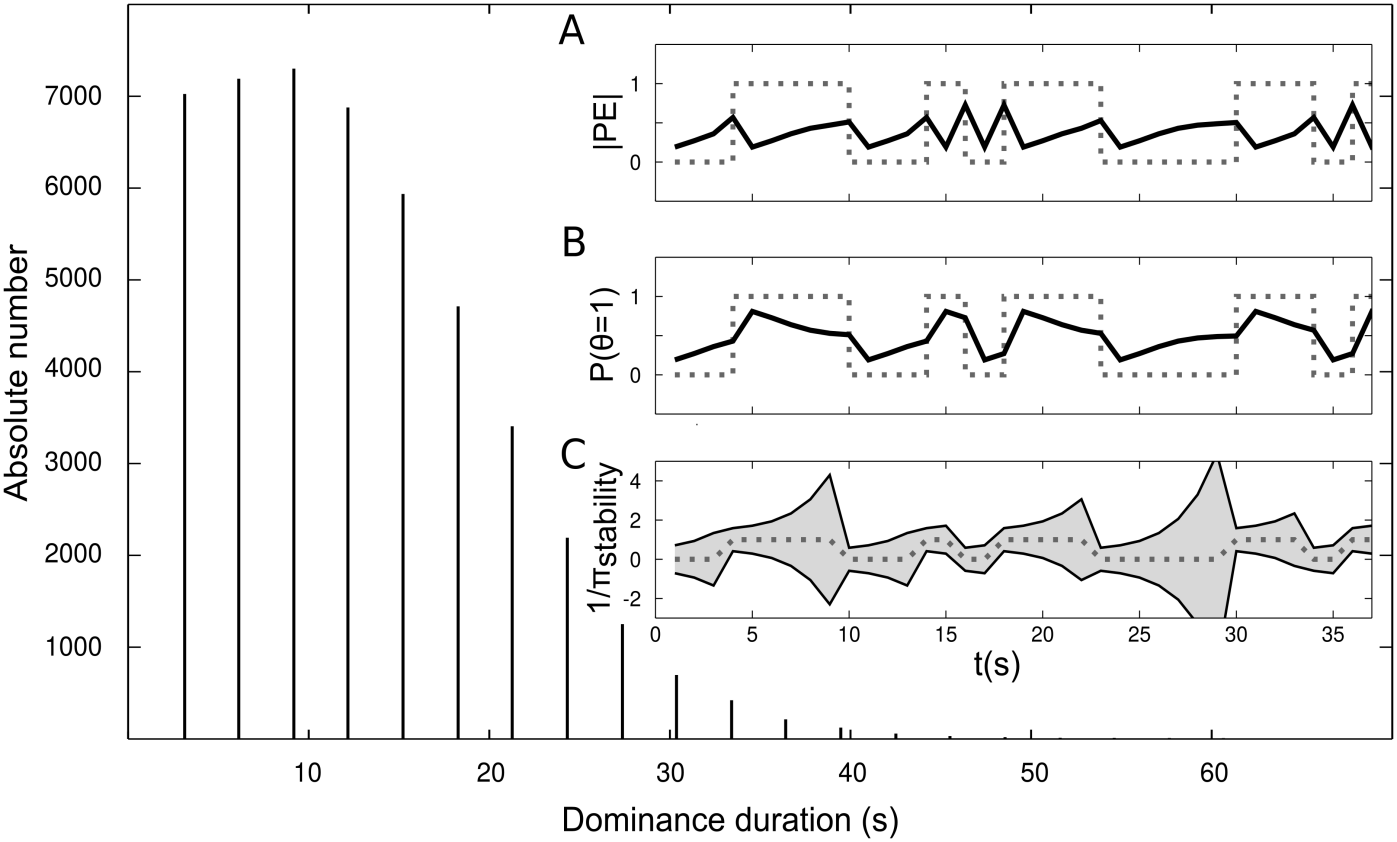
Simulating perceptual decisions during ambiguous stimulation. Data were simulated using *π*_*init*_ of 3.5 at a sampling rate of 0.33 Hz for a total of 6*10^5^ seconds. The distribution of phase durations followed a sharp rise and slow fall resembling a gamma-distribution. The insets A-C show simulated perceptual time-courses (grey dotted lines) next to updated model quantities (black solid lines). **A**: Prediction errors increase during a dominance phase and are reduced by perceptual transitions. **B**: Bistable perception can be conceived as resulting from subsequent sampling from a bimodal probability distribution [19], the weight of which is expressed by *P*(*θ* > 0.5). This weight is close to 0 or 1 at the beginning of a dominance phase (low transition probability) and gradually relaxes to 0.5 (high transition probability). **C**: The variance (inverse precision) of the prior distribution ‘perceptual stability’ increases as a consequence of prediction errors and is set to 1/*π*_*init*_ after a transition in perception.

The distribution of perceptual phase durations followed a sharp rise and slow fall (Fig 2) typical for bistable stimuli [29, 30]. Mean and median simulated phase durations were 10.40 and 10.00 seconds, closely matching the results from behavioural analysis (see *Modelling analysis of behavioural data*). As illustrated by exemplary time-courses of model parameters, the prediction error *PE* (Fig 2A) increases over time while one percept is dominant and is reduced once a new percept is adopted, reflecting the accumulation of evidence from the suppressed percept. The variance (1/*π*_*stability*_) of the prior ‘perceptual stability’ (Fig 2C) increases over a perceptual phase as a function of the prediction error. In line with the hypothesized role of prediction errors in driving perceptual transitions, the prediction error *PE* and, hence, the variance 1/*π*_*stability*_ are maximal when the posterior *P*(*θ* > 0.5) relaxes to 0.5 (Fig 2B), thereby increasing the probability of a new perceptual transition.

### Modelling analysis of behavioural data

To investigate whether our model is able to explain the dynamics of perceptual bistability in human observers, we fitted our model to behavioural data collected from 20 healthy participants during an fMRI experiment, in which participants viewed ambiguous and unambiguous (replay) versions of a rotating Lissajous stimulus. As reported previously, perceptual transitions occurred on average every 9.3 seconds in the ambiguity condition and neither block-by-block ratings nor debriefing after the experiment revealed differences in perceived appearance between the ambiguity and the replay condition [7].

We first performed a model comparison with other models that lacked the key conceptual elements of our model. By eliminating either the likelihood weight ‘stereodisparity’ or the prior ‘perceptual stability’ or both from the model, we constructed three additional models which we compared to our model using Random Effects Bayesian Model Selection. Our model (i.e. behavioural model 4) was identified as a clear winning model with a protected exceedance probability of 99.96%, demonstrating that the incorporation of both the likelihood weight ‘stereodisparity’ and the prior ‘perceptual stability’ best explained participants’ perception.

**Fig 3:**
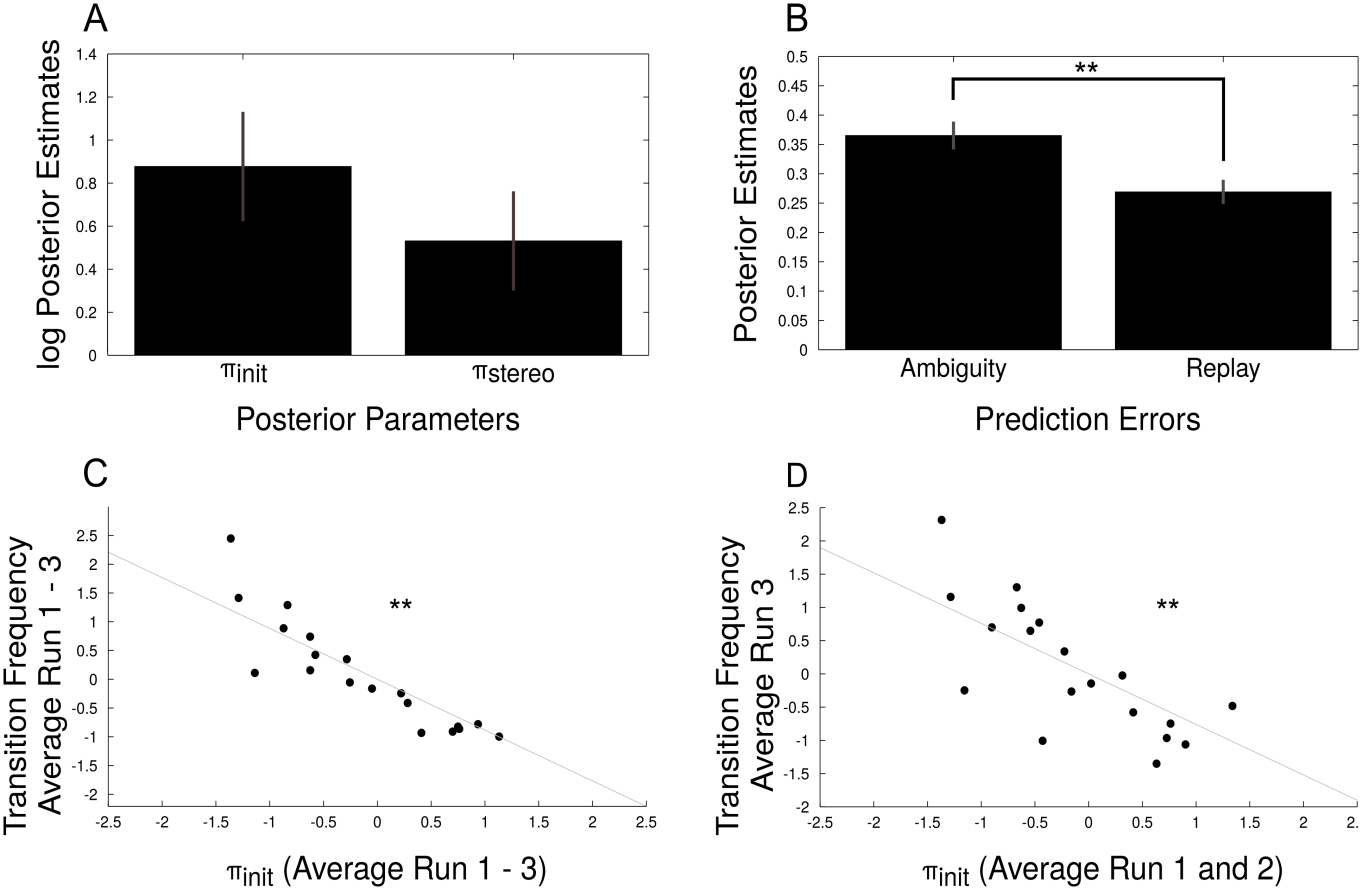
Posterior model parameters. **A**: The geometric mean (i.e. the arithmetic mean in log-space) of posterior *π*_*init*_ and *π*_*stereo*_, averaged across runs and participants, and standard error of the mean. **B**: Mean prediction errors averaged across runs and participants for ambiguous and replay blocks and standard error of the mean. Prediction errors were significantly decreased during replay stimulation (two-sample t-test, *p* < 10^−6^, *t*_19_ = 7.69). **C**: Average transition probabilities correlated significantly with average *π*_*init*_ for individual participants (*ρ* = −0.88, *p* < 10^−7^, Pearson correlation), providing a sanity check for model fit. **D**: Transition probabilities from run 3 were predictive of posterior *π*_*init*_ averaged over run 1 and 2. The significant Pearson correlation between the two independent measures (*ρ* = −0.76, *p* < 10^−4^) illustrates the predictive power of the model.

From this model, we extracted the parameters for *π*_*init*_ and *π*_*stereo*_ and averaged across runs and participants (Fig 3A). We predicted average prediction errors to be lower in replay as compared to the ambiguous condition, since the presented stereodisparity reduces the ambiguity left in the experimental display, and hence, the residual evidence for suppressed percept. Consistently, mean prediction errors were significantly higher in the ambiguous condition than in the replay condition (0.36 +/- 0.03 vs. 0.26 +/- 0.02, mean +/- s.e.m., *p* < 10^−6^, *t*_19_ = 7.06, two-sample t-test, Fig 3B), providing support for a correct implementation of our predictive coding model.

Given that *π*_*init*_ describes the strength of the initial stabilization after a switch in perception, we expected this parameter to be related to the frequency of perceptual transitions. In line with this, model parameter estimates *π*_*init*_ were negatively correlated with perceptual transition frequencies across participants (*ρ* = −0.88, *p* < 10^−7^, Pearson correlation, Fig 3C), providing a sanity check for model fit. Notably, this correlation was also significant when we correlated model parameter estimates for *π*_*init*_ averaged over run 1 and 2 with perceptual transition frequencies from run 3 (*ρ* = −0.76, *p* = 10^−4^, Fig 3D), corroborating that our model successfully accounted for observers’ perception evoked by an ambiguous stimulus.

We furthermore validated our approach by comparing our predictive coding model to established models of bistable perception from three different classes: oscillator models [1], attractor models [2] and intermediate models [3] (see Supplementary Methods in S2 Text). Data simulations indicated that all established models, similar to our predictive coding model, were able to produce spontaneous transitions in perception and a typical gamma-like distribution of perceptual phase durations (see Supplementary Results and Fig. A-C in S2 Text). Fitting of the behavioural data further showed that both the oscillator and the intermediate, similar to our predictive coding model, adequately accounted for the observers’ perceptual decisions during bistable perception (see Supplementary Results and Fig. D-I in S2 Text). In order to validate our approach, we conducted a Bayesian Model Comparison, which showed that our predictive coding model compared to these established models was best in explaining the behavioural data collected during this experiment (see Fig. J in S2 Text).

Please note that we did not carry out these analysis to demonstrate a superiority of our approach over these earlier models, which were initially conceived mainly for binocular rivalry and not for the prediction of behavioural responses during presentation of the Lissajous figure (a specific type of structure-from-motion stimulus). On the contrary, we aimed at probing the validity of our approach and tried to ascertain that the predictive coding approach was at least equivalent to existing models of bistable perception.

### Model-based fMRI analysis

One central aim of our study was to gain mechanistic insight into the neural processes underlying transition-related activity during bistable perception. We therefore performed both a model-based fMRI analysis suitable to identify the neural correlates of modelled prediction errors (‘PE model’), and, for the purpose of comparison, a conventional analysis (‘Conventional model’) dissociating between ambiguous and replay transitions as well as a ‘Block model’ accounting for effects of ambiguity per se.

To test the validity of these models, we first searched for voxels that were more active during visual stimulation as compared to baseline (‘overlaps vs. baseline’). For the ‘PE model’, this analysis revealed significant clusters (*p* < 0.05, FWE-corrected across the whole brain) bilaterally in middle occipital cortex (right: [39 -9 1], T = 10.21; left: [-30 -94 1], T = 13.30), in V5/hMT+ (right: [45 -70 1], T = 11.61; left: [-45 -73 4], T = 14.09), as well as in superior parietal cortex (right: [27 -49 58], T = 10.26; left: [-36 -46 -61], T = 8.62). The same analyses for the ‘Conventional model’ and the ‘Block model’ yielded virtually identical results (see Tables 2–4), confirming the comparability between all three models.

**Table 2:** ‘PE model’: Overlaps vs baseline.

Cluster	T	MNI			Region
**1**	T = 11.61	45	-70	1	R Middle Temporal Gyrus
	T = 10.94	30	-91	-5	R Inferior Occipital Gyrus
	T = 10.21	39	-79	1	R Middle Occipital Gyrus
**2**	T = 10.26	27	-49	58	R Superior Parietal Lobule
	T = 0.22	30	-46	55	R Postcentral Gyrus
	T = 8.93	21	-58	58	R Superior Parietal Lobule
**3**	T = 11.96	-27	-52	55	L Inferior Parietal Lobule
	T = 8.62	-36	-46	61	L Superior Parietal Lobule

**Table 3:** ‘Conventional model’: Overlaps vs baseline.

Cluster	T	MNI			Region
**1**	T = 11.64	42	-70	-2	R Middle Temporal Gyrus
	T = 10.92	30	-91	-5	R Inferior Occipital Gyrus
	T = 10.22	39	-79	1	R Middle Occipital Gyrus
**2**	T = 10.17	27	-52	61	R Superior Parietal Lobule
	T = 10.09	30	-49	58	R Superior Parietal Lobule
	T = 8.90	21	-58	58	R Superior Parietal Lobule
**3**	T = 11.82	-27	-52	55	L Inferior Parietal Lobule
	T = 8.35	-36	-46	61	L Superior Parietal Lobule

**Table 4:** ‘Block model’: Overlaps vs baseline.

Cluster	T	MNI			Region
**1**	T = 11.60	42	-70	-2	R Middle Temporal Gyrus
	T = 10.96	30	-91	-5	R Inferior Occipital Gyrus
	T = 10.26	39	-79	1	R Middle Occipital Gyrus
**2**	T = 10.19	27	-52	61	R Superior Parietal Lobule
	T = 10.10	30	-46	55	R Postcentral Gyrus
	T = 8.89	21	-58	58	R Superior Parietal Lobule
**3**	T = 11.78	-27	-52	55	L Inferior Parietal Lobule
	T = 8.36	-36	-46	61	L Superior Parietal Lobule

**Fig 4:**
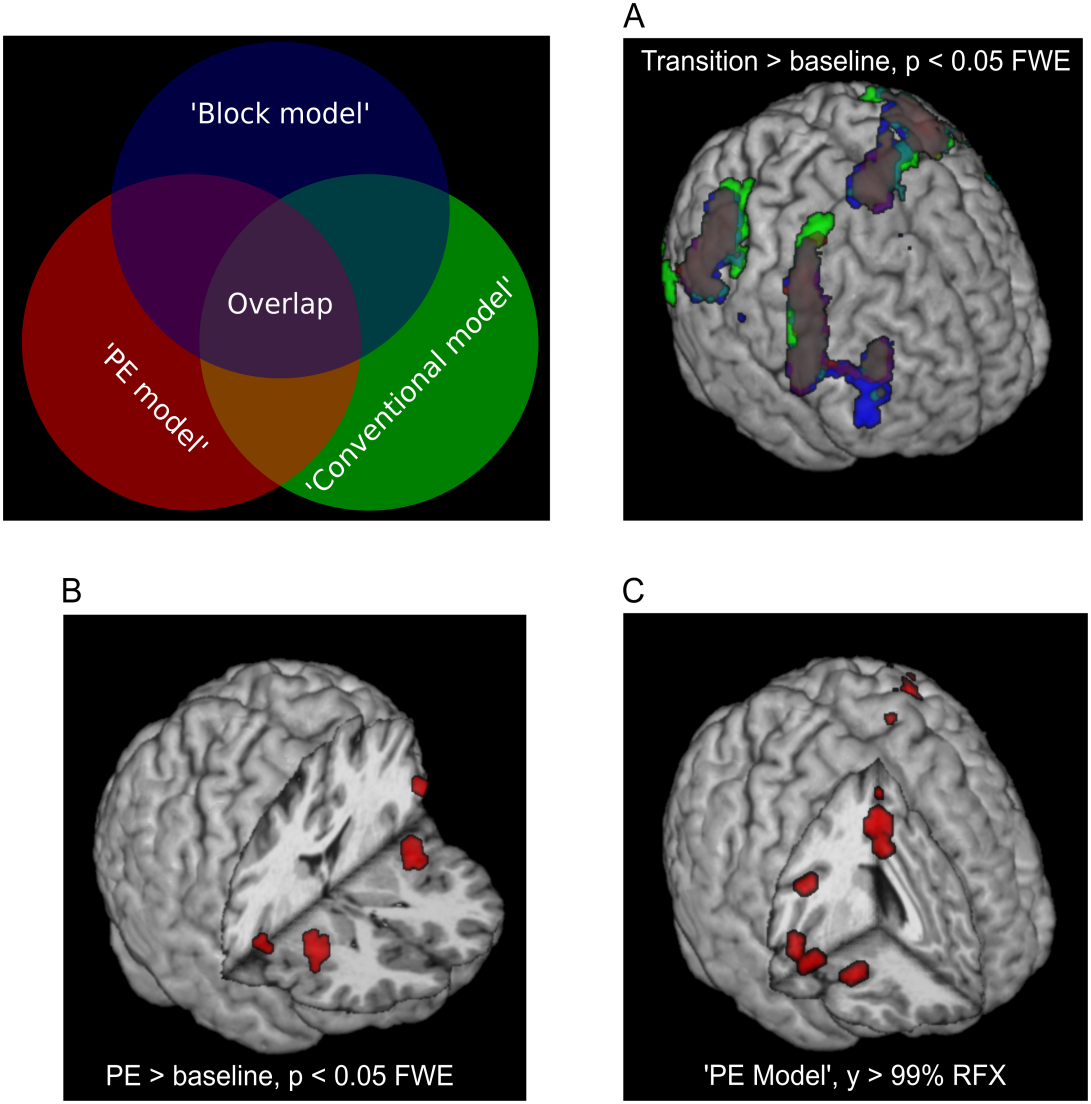
Model-based fMRI results from standard GLM (A, B) and PPM (C) analyses. GLMs are displayed using FWE correction at *p* < 0.05. For PPM results, we show voxels above an exceedance probability of 99% with a cluster size *n* > 10. **A**: 2-level contrast for ‘Transition vs. baseline’ showing activations left pre- and postcentral gyrus, right inferior frontal gyrus, right inferior parietal lobulus and right middle frontal gyrus with qualitatively equivalent results for all models. **B**: ‘PE vs. baseline’ (‘PE model’) yielded activations in bilateral insulae and inferior frontal gyri. We found no whole-brain correctable voxels for ‘Ambiguity vs. baseline’ (‘Block model’) nor for ‘Ambiguous vs. replay transitions’ (‘Conventional model’). **C**: Group exceedance probability maps with right insula, right inferior frontal gyrus, right posterior-medial frontal gyrus as well as left precentral gyrus showed strongest evidence for the ‘PE model’ as compared to the control models.

We then investigated which voxels were more active during perceptual transitions as compared to baseline (‘transitions vs. baseline’, Fig 4A): For the ‘PE model’, we found significant activations of motor-related areas in left precentral gyrus ([-36 -16 67], T = 12.23) extending to left postcentral gyrus ([-63 -19 25], T = 8.62) as well as significant clusters in regions previously associated with transition-related activity during bistable perception: right inferior frontal gyrus ([54 17 13], T = 7.96), right inferior parietal lobulus (54 -37 52, T = 9.32) and right middle frontal gyrus ([39 44 31], T = 7.57). Additional clusters were located in bilateral posterior-medial frontal gyrus (right: [6 2 67], T = 9.50; left: [-6 2 55], T = 12.63). Again, repeating this analysis for the ‘Block model’ and the ‘Conventional model’ yielded qualitatively very similar results as in the ‘PE model’ (see Tables 5–7), thereby providing further evidence for the validity and comparability of all three models.

**Table 5:** ‘PE model’: Transitions vs baseline.

Cluster	T	MNI			Region
**1**	T = 12.23	-36	-16	67	L Precentral Gyrus
	T = 8.74	-51	-28	43	L Inferior Parietal Lobule
	T = 8.28	-57	-28	34	L SupraMarginal Gyrus
**2**	T = 11.19	42	2	46	R Precentral Gyrus
	T = 9.73	42	8	40	R Middle Frontal Gyrus
	T = 7.71	15	5	13	R Caudate Nucleus
	T = 7.96	54	17	13	R IFG (p. Opercularis)
**3**	T = 12.63	-6	2	55	L Posterior-Medial Frontal
	T = 9.50	6	2	67	R Posterior-Medial Frontal
**4**	T = 9.42	60	-40	43	R SupraMarginal Gyrus
**5**	T = 6.70	42	26	-5	R Insula
**6**	T = 7.03	-18	-97	-8	L Inferior Occipital Gyrus
**7**	T = 6.50	-27	-88	-2	L Middle Occipital Gyrus

**Table 6:** ‘Conventional model’: Transitions vs baseline.

Cluster	T	MNI			Region
**1**	T = 14.73	-6	-1	55	L Posterior-Medial Frontal
	T = 12.88	-36	-16	67	L Precentral Gyrus
	T = 10.33	-42	-7	4	L Insula Lobe
**2**	T = 10.11	60	-40	43	R SupraMarginal Gyrus
	T = 9.57	51	-40	55	R Inferior Parietal Lobule
**3**	T = 7.97	42	44	28	R Middle Frontal Gyrus
**4**	T = 7.30	39	-46	40	R Inferior Parietal Lobule
**5**	T = 6.80	-21	-94	-8	L Inferior Occipital Gyrus
**6**	T = 6.82	33	-58	43	R Angular Gyrus

**Table 7:** ‘Block model’: Transitions vs baseline.

Cluster	T	MNI			Region
**1**	T = 14.71	-6	-1	55	L Posterior-Medial Frontal
	T = 12.58	-42	-22	58	L Postcentral Gyrus
	T = 12.57	-36	-16	67	L Precentral Gyrus
	T = 10.51	-42	-7	4	L Insula Lobe
	T = 9.65	42	8	37	R Middle Frontal Gyrus
**2**	T = 10.17	60	-40	43	R SupraMarginal Gyrus
	T = 9.57	51	-40	55	R Inferior Parietal Lobule
**3**	T = 6.89	33	-61	43	R Angular Gyrus
**4**	T = 6.71	-18	-97	-8	L Inferior Occipital Gyrus

To formally test whether the modelled prediction error explains the BOLD signal better than the conventional comparison of ambiguous with replay perceptual switches (‘Conventional model’), or the mere ambiguity of the visual display (the ‘Block model’), we performed a PPM analysis [22] to compute voxel-wise exceedance probability maps for the ‘PE model’ against the ‘Conventional model’ and the ‘Block model’ (Fig 4C). We restricted this analysis to areas of the fronto-parietal cortex, which be delineated by intersecting the statistical-parametric maps for ‘transitions vs. baseline’ thresholded at *p* < 0.05 FWE for all three models considered. Remarkably, when applying a conservative threshold of an exceedance probability of *γ* = 99% and a cluster size of *n* > 10 voxels, we found clusters in right insula ([39 26 -2]) and right inferior frontal gyrus ([51 14 1]) to show strong evidence for the ‘PE model’ as compared to the ‘Block model’ and the ‘Conventional model’. Additional clusters were located in right posterior medial frontal gyrus ([6 5 49]) as well as left precentral gyrus ([-36 -16 52]).

Conversely, for the exceedance probability map of the ‘Conventional model’ compared against ‘Block model’ and ‘PE model’, no voxels survived the conservative threshold used in the main experiment. For the exceedance probability map of the ‘Block model’ compared against the ‘Conventional model’ and ‘PE model’, we found clusters in bilateral inferior parietal lobule at an exceedance probability of 99% and a cluster size > 10.

For our central analysis aimed at identifying the neural correlates of modelled prediction errors, we searched for voxels in which BOLD activity was related to the parametric modulator of the ‘PE model’ that encoded prediction error trajectories from our Bayesian model of bistable perception (Fig 4B). We found significant clusters (*p* < 0.05, FWE-corrected across the whole brain) in bilateral insulae (right: [33 23 7], T = 7.24; left: [-30 20 10], T = 7.88) and bilateral inferior frontal gyri (right: [51 8 10], T = 6.89; left: [- 54 2 22], T = 6.67). These regions are located in close anatomical proximity to frontal regions previously suggested to mediate perceptual transitions in bistable perception [4, 5, 7].

**Fig 5:**
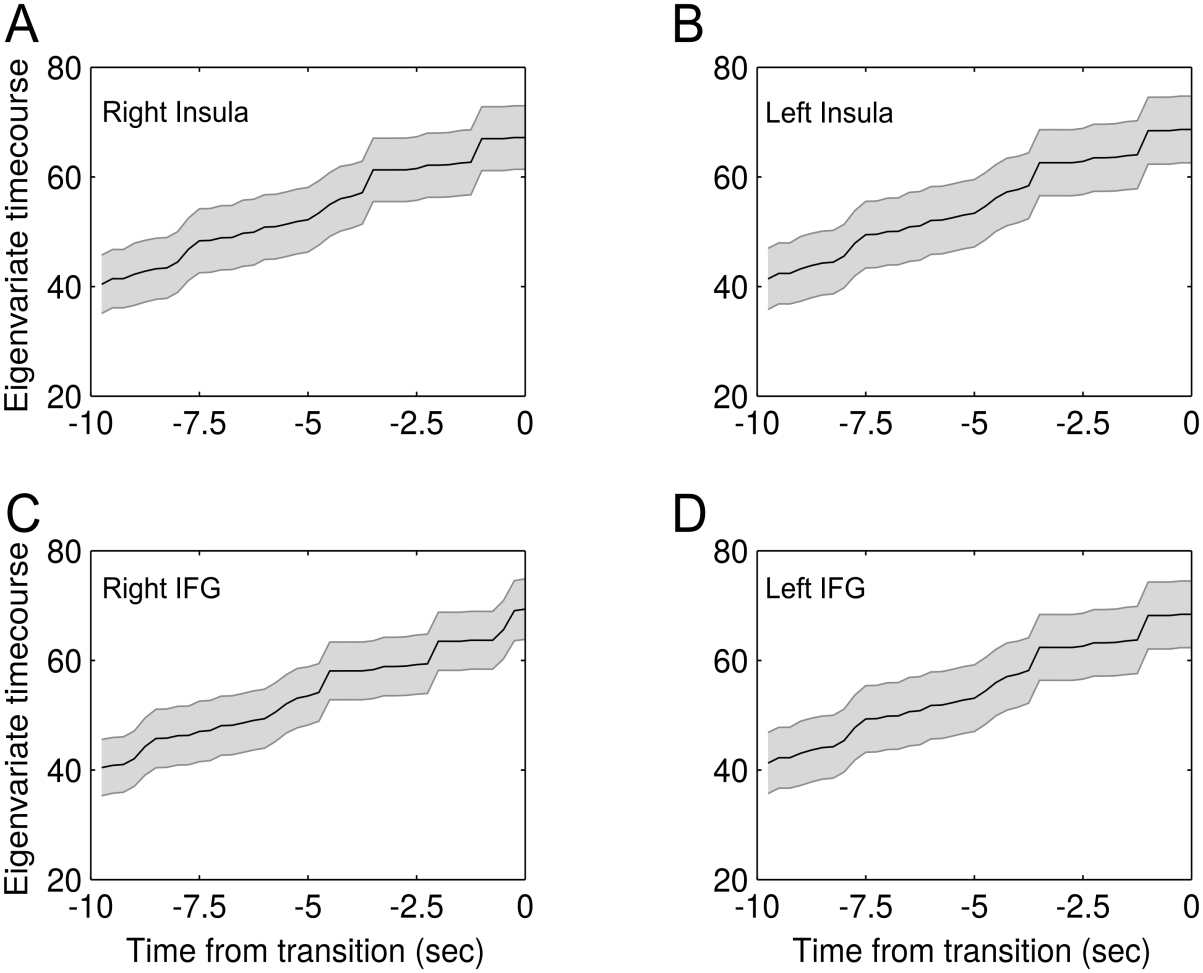
Average eigenvariate time-courses. For visualization, we extracted eigenvariate time-courses from right insula, left insula, right IFG and left IFG (A–D), aligned all phase durations to the timepoint of the upcoming perceptual transition and averaged within and across observers. In analogy to modelled prediction error trajectories, mean eigenvariate time-courses (± standard error of the mean) showed a gradual increase towards a transition in perception.

In order to further visualize the correlation between modelled prediction error and BOLD activity, we extracted eigenvariate time-courses from right insula, left insula, right IFG as well as left IFG and averaged across perceptual phase durations and observers. As expected, these time-courses showed a gradual increase towards a transition in perception (Fig 5), nicely mirroring the build-up of prediction error during a perceptual phase.

## Discussion

In this work, we present a Bayesian predictive coding model for bistable perception, which rests on the basic assumption that prediction errors are elicited by the unexplained alternative interpretation of an ambiguous stimulus and represent the driving force behind perceptual transitions during bistable perception. We found that this model is able to reproduce key temporal characteristics of human bistable perception and that it explains observers’ behaviour during a bistable perception experiment. Our central finding shows that modelled prediction errors correlate with BOLD activity in bilateral insulae and bilateral inferior frontal gyrus. Remarkably, our PPM analysis revealed that modelled prediction errors best accounted for BOLD activity as compared to mere occurrence of endogenous perceptual transitions or ambiguity of the visual display in these frontal regions. Hence, our current results suggest that prediction errors might provide the mechanistic basis for perceptual switching in bistable perception and offer a novel interpretation of frontal activity in bilateral insulae as well as the right inferior frontal gyrus during bistable perception.

The functional significance of enhanced frontal brain activity for transitions during bistability as compared to an unambiguous control condition is a matter of ongoing debate: Some authors proposed that non-sensory higher-level brain regions are actively implicated in resolving the perceptual conflict during bistable perception, thus mediating perceptual transitions [4, 31, 5, 11, 7]. Others have argued that perceptual conflicts are resolved primarily in sensory brain areas and that activity in frontal and parietal regions reflects the registration and/or report of perceptual transitions, rather than their cause [6, 8, 10]. For a detailed discussion of this debate, see “Brascamp, Sterzer, Blake and Knapen, Multistable perception and the role of frontoparietal cortex in perceptual inference, Annual Review of Psychology, in press.”

Here, we provide further evidence for an active implication of frontal regions in bistable perception by functionally relating these regions to a prediction error signal. Hence, our work is in line with hybrid models that suggest bistable perception to arise from an interplay between lower-level sensory and higher-level non-sensory areas [32, 12, 11]. In this context, it might be speculated that prediction errors are computed in frontal regions based on feedforward signals from visual and parietal cortex; and that these prediction errors, in turn, modulate activity in visual cortex via feedback signals.

In addition to the prediction error, the stability prior represents an essential element of our predictive coding model of bistable perception, since its initial precision determines the frequency of perceptual transitions. The notion of such a stability prior is supported by experimental work on serial dependence in visual perception: In an orientation-judgement task, [33] showed that perceived orientation was biased by recently observed stimuli and reasoned that the visual system might use past experiences as predictors of present perceptual decisions, thereby incorporating representations of the continuity of the visual environment. Corroborating these results in a fMRI experiment, [34] found that orientation signals in early visual cortex were biased towards previous perceptual decisions. At this point, however, we can only speculate about the neural correlates of the stability prior from our model: In recent work on the role of parietal cortex in bistable perception, [35] and [9] have proposed a functional segregation of the superior parietal lobulus (SPL), which they deduced from differential effects of grey matter volume on perceptual dominance durations and analyses of effective connectivity on the basis of fMRI. By interpreting their results in a Bayesian framework, the authors argued that posterior SPL might represent a prediction error, while the anterior SPL would entertain a perceptual prediction.

A key advantage of our predictive coding model of bistable perception is that it allows us to treat ambiguous and replay stimulation within the same framework. By formalizing the disambiguating factor as a weight on a bimodal likelihood distribution, such models can be used to investigate perceptual transitions under varying degrees of ambiguity, thus dissolving the artificial dichotomy between the two conditions. Hence, such models provide a new perspective on how the brain might resolve perceptual conflicts despite the ambiguity inherent in every sensory signal and offer a generic tool for quantifying the contribution of different contextual factors on perceptual outcomes.

The major strength of predictive coding models for bistable perception, however, lies in their ability to parsimoniously link different levels in the description of perceptual dynamics in ambiguous visual environments: On a computational level, prediction errors constitute the driving force behind perceptual transitions and are substantially reduced by additional sensory information (such as stereodisparity) during replay. On a neural level, casting frontal activity during rivalry in terms of prediction error signals nicely relates to increased transition-related activity [4, 5, 9] and connectivity [7]. On a theoretical level, viewing perceptual transitions as means of reducing prediction errors places bistable perception in the context of Bayesian theories of the brain [16, 36, 27, 37], and in particular the free-energy principle [13]. According to the latter, agents strive for a reduction of their model’s free energy, which translates onto a minimization of squared prediction errors in predictive coding schemes. When sensory information is constantly ambiguous, one possibility to reduce free energy is to update beliefs about the world, which ultimately corresponds to the adoption of a new percept.

However, given that the Lissajous differs in some aspects from other types of bistable stimuli, one has to consider important limitations regarding the generalization of our findings: While being physically ambiguous for all angles of rotation, transitions almost exclusively occur at overlapping stimulus configurations, which is similar to the behaviour of some types of random dot kinematograms [26] or intermittent presentation of bistable stimuli [38] and accompanied by a reduced incidence of mixed percepts or incomplete transitions. Since these phenomena are present in many other forms of bistable perception and significantly affect frontoparietal activity during perceptual transitions [6], our current imaging results can only be interpreted in relation to the specific stimulus used here.

A similar limitation applies to the behavioural modelling presented in this manuscript: Previous work on computational modelling of bistable perception has focused on a variety of mechanisms at the heart of spontaneous perceptual transitions: Oscillator models have focused on mutual inhibition between two competing neuronal populations combined with slow adaptation of the currently dominant population [1]. [39] have studied the differential effects of short and long interruptions in intermittent bistable perception for binocular rivalry and structure-from-motion and presented a model based on adaptive processes, cross-inhibition and neural baseline levels. Importantly, this model also accounts for the possibility of voluntary control via attentional processes interacting with early processing stages.

Alternative approaches view noise as the underlying cause of perceptual transitions [2]. Importantly, models belonging to this class have also taken account of the aforementioned mixed percepts and incomplete transitions during binocular rivalry [40].

Further models have related transitions in perception to a combination of adaptation and noise [3]. In this vein, [41] have argued for a neurodynamic mechanism at the bifurcation between adaptation- and noise-driven processes to be the basis for perceptual transitions during binocular rivalry.

The majority of the models mentioned above has been developed for continuous presentation of binocular rivalry or ambiguous structure-from-motion, while [39] have also studied paradigms with intermittent presentation. As noted above, such stimuli differ significantly from the Lissajous figure used in our current study, which shares aspects with intermittent stimulation due to the existence of overlapping configurations facilitating transitions in perception. Hence, future theoretical and empirical work is needed to probe our modelling approach on paradigms such as binocular rivalry and ambiguous structure-from-motion for both continuous and intermittent presentation and to extend the predictive coding model in order to account for top-down attentional control as well as interactions at earlier processing stages.

Taken together, our current work provides theoretical and empirical evidence across different levels for a driving role of prediction errors in bistable perception, thereby shedding new light on an ongoing debate about the neural mechanisms underlying bistable perception and, more generally, opening up a novel computational perspective on the mechanisms governing perceptual inference.

## Supporting information

S1 Text Mathematical appendix. The appendix contains a detailed mathematical description of our modelling procedures.
(PDF)

S2 Text Validation against established models of bistable perception. In this supplement, we provide a validation of our modelling approach against established models of bistable perception based on adaptation and inhibition [1], noise [2] and an intermediate model [3].
(PDF)

S1 Video Example of a full rotation of the specific Lissajous figure used in this experiment. (WMV)

## References

[1] Wilson HR. Minimal physiological conditions for binocular rivalry and rivalry. Vision research. 2007;47(21):2741–50. 10.1016/j.visres.2007.07.007 17764714

[2] Moreno-Bote R, Rinzel J, Rubin N. Noise-Induced Alternations in an Attractor Network Model of Perceptual Bistability. Journal of Neurophysiology. 2007;98(3):1125–1139. 10.1152/jn.00116.2007 17615138 PMC2702529

[3] Lehky SR. An astable multivibrator model of binocular rivalry. Perception. 1988;17(2):215–28. 10.1068/p170215 3067209

[4] Lumer ED, Friston KJ, Rees G. Neural correlates of perceptual rivalry in the human brain. Science (New York, NY). 1998;280(5371):1930–4. 10.1126/science.280.5371.1930 9632390

[5] Sterzer P, Kleinschmidt A. A neural basis for inference in perceptual ambiguity. Proceedings of the National Academy of Sciences of the United States of America. 2007;104(1):323–8. 10.1073/pnas.0609006104 17190824 PMC1765459

[6] Knapen T, Brascamp J, Pearson J, van Ee R, Blake R. The Role of Frontal and Parietal Brain Areas in Bistable Perception. Journal of Neuroscience. 2011;31(28):10293–10301. 10.1523/JNEUROSCI.1727-11.2011 21753006 PMC3146344

[7] Weilnhammer VA, Ludwig K, Hesselmann G, Sterzer P. Frontoparietal cortex mediates perceptual transitions in bistable perception. The Journal of neuroscience: the official journal of the Society for Neuroscience. 2013;33(40):16009–15. 10.1523/JNEUROSCI.1418-13.2013 24089505 PMC6618467

[8] Frässle S, Sommer J, Jansen A, Naber M, Einhäuser W. Binocular rivalry: frontal activity relates to introspection and action but not to perception. The Journal of neuroscience: the official journal of the Society for Neuroscience. 2014;34(5):1738–47. 10.1523/JNEUROSCI.4403-13.2014 24478356 PMC6827584

[9] Megumi F, Bahrami B, Kanai R, Rees G. Brain activity dynamics in human parietal regions during spontaneous switches in bistable perception. NeuroImage. 2015;107:190–7. 10.1016/j.neuroimage.2014.12.018 25512040 PMC4306523

[10] Brascamp J, Blake R, Knapen T. Negligible fronto-parietal BOLD activity accompanying unreportable switches in bistable perception. Nature neuroscience. 2015;18(11):1672–1678. 10.1038/nn.4130 26436901 PMC4603386

[11] Sterzer P, Kleinschmidt A, Rees G. The neural bases of multistable perception. Trends in cognitive sciences. 2009;13(7):310–8. 10.1016/j.tics.2009.04.006 19540794

[12] Hohwy J, Roepstorff A, Friston K. Predictive coding explains binocular rivalry: an epistemological review. Cognition. 2008;108(3):687–701. 10.1016/j.cognition.2008.05.010 18649876

[13] Friston K. The free-energy principle: a unified brain theory? Nature reviews Neuroscience. 2010;11(2):127–38. 10.1038/nrn2787 20068583

[14] Clark A. Whatever next? Predictive brains, situated agents, and the future of cognitive science. The Behavioral and brain sciences. 2013;36(3):181–204. 10.1017/S0140525X12000477 23663408

[15] Rao RP, Ballard DH. Predictive coding in the visual cortex: a functional interpretation of some extra-classical receptive-field effects. Nature neuroscience. 1999;2(1):79–87. 10.1038/4580 10195184

[16] Lee TS, Mumford D. Hierarchical Bayesian inference in the visual cortex. Journal of the Optical Society of America A, Optics, image science, and vision. 2003;20(7):1434–48. 10.1364/JOSAA.20.001434 12868647

[17] Friston K. A theory of cortical responses. Philosophical transactions of the Royal Society of London Series B, Biological sciences. 2005;360(1456):815–36. 10.1098/rstb.2005.1622 15937014 PMC1569488

[18] Mathys CD, Lomakina EI, Daunizeau J, Iglesias S, Brodersen KH, Friston KJ, et al Uncertainty in perception and the Hierarchical Gaussian Filter. Frontiers in human neuroscience. 2014;8:825 10.3389/fnhum.2014.00825 25477800 PMC4237059

[19] Sundareswara R, Schrater PR. Perceptual multistability predicted by search model for Bayesian decisions. Journal of vision. 2008;8(5):12.1–19. 10.1167/8.5.12 18842083

[20] Stephan KE, Penny WD, Daunizeau J, Moran RJ, Friston KJ. Bayesian model selection for group studies. NeuroImage. 2009;46(4):1004–17. 10.1016/j.neuroimage.2009.03.025 19306932 PMC2703732

[21] O’Doherty JP, Hampton A, Kim H. Model-based fMRI and its application to reward learning and decision making. Annals of the New York Academy of Sciences. 2007;1104(1):35–53. 10.1196/annals.1390.022 17416921

[22] Rosa MJ, Bestmann S, Harrison L, Penny W. Bayesian model selection maps for group studies. NeuroImage. 2010;49(1):217–24. 10.1016/j.neuroimage.2009.08.051 19732837 PMC2791519

[23] Brainard DH. The Psychophysics Toolbox. Spatial vision. 1997;10(4):433–6. 10.1163/156856897X00357 9176952

[24] Schurger A. A very inexpensive MRI-compatible method for dichoptic visual stimulation. Journal of neuroscience methods. 2009;177(1):199–202. 10.1016/j.jneumeth.2008.09.028 18973774

[25] Weilnhammer VA, Sterzer P, Hesselmann G. Perceptual Stability of the Lissajous Figure Is Modulated by the Speed of Illusory Rotation. PloS one. 2016;11(8):e0160772 10.1371/journal.pone.0160772 27560958 PMC4999232

[26] Pastukhov A, Vonau V, Braun J. Believable change: bistable reversals are governed by physical plausibility. Journal of vision. 2012;12(1). 10.1167/12.1.17 22267054

[27] Friston KJ, Stephan KE. Free-energy and the brain. Synthese. 2007;159(3):417–458. 10.1007/s11229-007-9237-y 19325932 PMC2660582

[28] Eickhoff SB, Stephan KE, Mohlberg H, Grefkes C, Fink GR, Amunts K, et al A new SPM toolbox for combining probabilistic cytoarchitectonic maps and functional imaging data. NeuroImage. 2005;25(4):1325–35. 10.1016/j.neuroimage.2004.12.034 15850749

[29] Levelt WJM. Note on the Distribution of Dominance Times in Binocular Rivalry. British Journal of Psychology. 1967;58(1-2):143–145. 10.1111/j.2044-8295.1967.tb01068.x 5582864

[30] Logothetis NK, Leopold DA, Sheinberg DL. What is rivalling during binocular rivalry? Nature. 1996;380(6575):621–4. 10.1038/380621a0 8602261

[31] Leopold DA, Logothetis NK. Multistable phenomena: changing views in perception. Trends in Cognitive Sciences. 1999;3(7):254–264. 10.1016/S1364-6613(99)01332-7 10377540

[32] Tong F, Meng M, Blake R. Neural bases of binocular rivalry. Trends in cognitive sciences. 2006;10(11):502–11. 10.1016/j.tics.2006.09.003 16997612

[33] Fischer J, Whitney D. Serial dependence in visual perception. Nat Neurosci. 2014;17(5):738–743. 10.1038/nn.3689 24686785 PMC4012025

[34] St John-Saaltink E, Kok P, Lau HC, de Lange FP. Serial Dependence in Perceptual Decisions Is Reflected in Activity Patterns in Primary Visual Cortex. Journal of Neuroscience. 2016;36(23):6186–6192. 10.1523/JNEUROSCI.4390-15.2016 27277797 PMC6604889

[35] Kanai R, Carmel D, Bahrami B, Rees G. Structural and functional fractionation of right superior parietal cortex in bistable perception. Current Biology. 2011;21(3):R106–R107. 10.1016/j.cub.2010.12.009 21300270 PMC3084447

[36] Knill DC, Pouget A. The {Bayesian} brain: the role of uncertainty in neural coding and computation. Trends Neurosci. 2004;27(12):712–719. 10.1016/j.tins.2004.10.007 15541511

[37] Hohwy J. Attention and conscious perception in the hypothesis testing brain. Frontiers in psychology. 2012;3:96 10.3389/fpsyg.2012.00096 22485102 PMC3317264

[38] Pearson J, Brascamp J. Sensory memory for ambiguous vision. Trends Cogn Sci (Regul Ed). 2008;12(9):334–341. 10.1016/j.tics.2008.05.006 18684661

[39] Klink PC, van Ee R, Nijs MM, Brouwer GJ, Noest AJ, van Wezel RJA. Early interactions between neuronal adaptation and voluntary control determine perceptual choices in bistable vision. Journal of Vision. 2008;8(5):16 10.1167/8.5.16 18842087

[40] Brascamp JW, van Ee R, Noest AJ, Jacobs RHAH, van den Berg AV, R B. The time course of binocular rivalry reveals a fundamental role of noise. Journal of Vision. 2006;6(11):8–8. 10.1167/6.11.8 17209732

[41] Panagiotaropoulos TI, Kapoor V, Logothetis NK, Deco G. A Common Neurodynamical Mechanism Could Mediate Externally Induced and Intrinsically Generated Transitions in Visual Awareness. PLoS ONE. 2013;8(1):e53833 10.1371/journal.pone.0053833 23349748 PMC3547944

[42] Weilnhammer VA, Ludwig K, Sterzer P, Hesselmann G. Revisiting the Lissajous figure as a tool to study bistable perception. Vision research. 2014;98:107–12. 10.1016/j.visres.2014.03.013 24718018

