# Supplementary material for: A predictive coding account of bistable perception - a model-based fMRI study
Source: PLoS Comput Biol. 2017 May 15;13(5):e1005536. doi: 10.1371/journal.pcbi.1005536 (PMC5448813; doi:10.1371/journal.pcbi.1005536)
Supplement: S1 Text — The appendix contains a detailed mathematical description of our modelling procedures. (PDF) [file pcbi.1005536.s001.pdf]

A PREDICTIVE CODING ACCOUNT OF BISTABLE PERCEPTION - A MODEL-BASED FMRI STUDY

Mathematical Appendix

As an example for an ambiguous stimulus, we use the Lissajous figure that - due to its ambiguous depth structure - is alternately perceived as a clockwise (as viewed fom above, i.e. movement of the front surface to the left) and counter-clockwise (vice versa) rotating object (see also supplementary video).

From a predictive coding perspective, the brain entertains and inverts a generative model of how sensory data are caused. In our case, the sensory environment is constrained to objects which rotate either clockwise or counter-clockwise, while direction of rotation changes at a specific frequency. In analogy, our model represents a generative model of how potentially ambiguous sensory data are caused by objects in the sensory environment, while the frequency of changes is governed by an implicit prior for stability.

The inversion of this model is based on sensory information $\mu_{stereo}(t)$ and the prediction of the perceived direction of rotation $y(t)$. It allows for the estimation of model parameters (i.e. the initial precision of the stability prior π_{init}; the precision of sensory stimulation π_{stereo}, the inverse decision temperature ζ). These model parameters govern the updates in model quantities (i.e. the mean and precision of the stability prior $\mu_{stability}$ and $\pi_{stability}$; the mean and precision of the joint prior distribution μ_m and π_m; the probability of perceiving counter-clockwise rotation

$P(\theta > 0.5)$; the predicted perceptual response $y_{predicted}$). See Table 7 for a list of model parameters and model quantities.

As new perceptual decisions on the rotation of the Lissajous figure are made almost exclusively at overlapping stimulus configurations ('overlaps', [1, 2]). we can convert the continuous time-course of stimulus presentation to discrete timepoints t of 'overlaps'. Accordingly, the sampling rate of our model is given by the frequency at which 'overlaps' occur and depends on the rotational speed of the Lissajous figure.

At each timepoint t, the two alternative visual percepts are predicted on the basis of a posterior probability distbution over θ:

$$\theta = \begin{cases} > 0.5 : & \rightarrow \quad (rotation) \\ < 0.5 : & \leftarrow \quad (rotation) \end{cases} \tag{1}$$

Participants responded with button-presses indicating the current visual percept:

$$y(t) = \begin{cases} 1 : & \rightarrow \quad (rotation) \\ 0 : & \leftarrow \quad (rotation) \end{cases} \tag{2}$$

Similar to other ambiguous stimuli, the Lissajous figure can be disambiguated by additional cues - e.g. by stereodisparity between the two eyes - in order to create a 'replay' condition.

Here, perception is forced to alternate between a clockwise and counter-clockwise rotating stimulus with a similar time-course as during bistable perception. Hence, we formalize the sensory information in both replay and ambiguity by the Gaussian distribution 'stereodisparity' ($\mathcal{N}(\mu_{stereo}, \pi_{stereo}^{-1})$), which is used as a weight on a bimodal likelihood distribution (see Equation 7 - 10, [3]). The mean of this distribution μ_{stereo} is given by the direction of disambiguation at timepoint t:

$$\mu_{stereo}(t) = \begin{cases} 1: & \rightarrow \quad (disambiguation) \\ 0.5: & \leftrightarrow \quad (ambiguous) \\ 0: & \leftarrow \quad (disambiguation) \end{cases} \tag{3}$$

Its precision π_{stereo} (the inverse of its variance) encodes the strength of disambiguation, and is either chosen as a free parameter or fixed to 0 (thereby eliminating a contribution of a disambiguation on the prediction of perceptual decisions).

Furthermore, we hypothesize that the current percept represents an (implicit) prior belief contributing to the stability of visual perception, which is given by the Gaussian distribution 'perceptual stability' ($\mathcal{N}(\mu_{stabiliy}, \pi_{stability}^{-1})$). The mean of this prior $\mu_{stability}$ at timepoint t is defined by the current percept as indicated by the participant at the preceding overlap:

$$\mu_{stability}(t) = y(t-1) \tag{4}$$

In turn, the impact of this prior distribution on visual perception is reflected by its precision $\pi_{stability}$. Central to our model of bistable perception, we allow this precision to be affected by a prediction error signal.

If a new perceptual decision was made at the preceding timepoint, $\pi_{stability}(t)$ is set to the initial perceptual precision π_{init}:

$$\pi_{stability}(t = t_0) = \pi_{init} \tag{5}$$

This initial perceptual precision π_{init} represents the strength of an initial perceptual stabilization following a perceptual transition and can be chosen as free parameter or fixed to 0 (thus eliminating the stability prior from the model).

In all other cases, $\pi_{stability}(t)$ is calculated by updating the perceptual precision of the preceding timepoint $\pi_{stability}(t-1)$ with the prediction error of the preceding timepoint $PE(t-1)$:

$$\pi_{stability}(t \neq t_0) = \pi_{stability}(t-1) * exp(-|PE(t-1)|) \tag{6}$$

To compute a posterior distribution, we combine the prior distribution 'perceptual stability' (parameterized by $\mu_{stability}$ and $\pi_{stability}$) with the 'stereodisparity'-weight of the likelihood (parameterized by μ_{stereo} and π_{stereo}) into a joint distribution m:

$$\pi_m = \pi_{stereo} + \pi_{stability} \tag{7}$$

$$\mu_m = \frac{\pi_{stereo} * \mu_{stereo} + \pi_{stability} * \mu_{stability}}{\pi_m} \tag{8}$$

The joint distribution m is used as weight on a bimodal likelihood distribution [3] in order to calculate the density ratio of the posterior for the two peak locations $\theta_0 = 0$ and $\theta_1 = 1$:

$$r = \frac{P(\theta < 0.5)}{P(\theta > 0.5)} = exp(\frac{(\theta_0 - \mu_m)^2 - (\theta_1 - \mu_m)^2}{2 * \pi_m^{-1}}) \tag{9}$$

$$P(\theta > 0.5) = \frac{1}{r + 1} \tag{10}$$

Please note that it is an arbitrary choice which of the two directions we consider, as the two posterior probabilities $P(\theta > 0.5)$ and $P(\theta < 0.5)$ sum up to 1.

By applying a unit sigmoid function parametrized by the inverse decision temperature ζ to the

posterior probability of counter-clockwise rotation $P(\theta > 0.5)(t)$, we predict the participants response $y(t)$, which represents the basis for the optimization of model parameters:

$$y_{predicted}(t) = \frac{P(\theta > 0.5)^{\zeta}}{P(\theta > 0.5)^{\zeta} + (1 - P(\theta > 0.5))^{\zeta}} \tag{11}$$

Most importantly, we use the difference between the current percept $y(t)$ as indicated by the participant and the posterior probability of counter-clockwise rotation $P(\theta > 0.5)(t)$ to calculate a prediction error $PE(t)$ that represents the residual evidence in favour of the suppressed percept:

$$PE(t) = y(t) - P(\theta > 0.5)(t) \tag{12}$$

It is noteworthy that the inclusion of a stereodisparity weight allows us to treat both ambiguity and replay within the same framework: The prediction error $PE(t)$ is also computed in the replay condition and shows similar temporal dynamics in the replay condition as in the ambiguity condition. The stereodisparity weight ($\mu_{stereo} \neq 0.5$), however, renders the posterior probability $P(\theta > 0.5)$ more similar to the currently induced percept $y(t)$ as compared to the ambiguity condition, where the stereodisparity weight (mean $\mu_{stereo} = 0.5$) is uninformative with respect

to the current percept. Hence, the prediction error $PE(t)$ (the difference between $P(\theta > 0.5)$ and $y(t)$) is expected to be smaller in replay as compared to ambiguity.

References

1. Weilnhammer VA, Ludwig K, Hesselmann G, Sterzer P. Frontoparietal cortex mediates perceptual transitions in bistable perception. The Journal of neuroscience : the official journal of the Society for Neuroscience. 2013;33(40):16009–15. doi:10.1523/JNEUROSCI.1418-13.2013.

2. Weilnhammer VA, Ludwig K, Sterzer P, Hesselmann G. Revisiting the Lissajous figure as a tool to study bistable perception. Vision research. 2014;98:107–12. doi:10.1016/j.visres.2014.03.013.

3. Sundareswara R, Schrater PR. Perceptual multistability predicted by search model for Bayesian decisions. Journal of vision. 2008;8(5):12.1–19. doi:10.1167/8.5.12.
