# Supplementary material for: A predictive coding account of bistable perception - a model-based fMRI study
Source: PLoS Comput Biol. 2017 May 15;13(5):e1005536. doi: 10.1371/journal.pcbi.1005536 (PMC5448813; doi:10.1371/journal.pcbi.1005536)
Supplement: S2 Text — In this supplement, we provide a validation of our modelling approach against established models of bistable perception based on adaptation and inhibition [1], noise [2] and an intermediate model [3]. (PDF) [file pcbi.1005536.s002.pdf]

A PREDICTIVE CODING ACCOUNT OF BISTABLE PERCEPTION - A MODEL-BASED FMRI STUDY

Supplementary Information

Introduction

To further validate our modeling approach, we aimed to compare our predictive coding model to established models of bistable perception. We chose three exemplary models belonging to three different model classes: ”oscillator models”, ”noise-driven atttractor models” and ”intermediate models”.

Alternations in bistable perception have been related to the interaction between two ore more competing neuronal populations coding for the alternative percepts, where the currently dominant population is subject to adaptation [1, 2, 3]. Here, we chose the model proposed by [4] as a classical representative of such ”oscillator” models.

Alternative approaches have considered noise to be the driving force behind transitions in bistable perception. In this framework, the alternative percepts during bistable perception have been conceived as stable states in a a dynamic neural network, while internal noise (e.g. variability in vesicular release, spiking rate or neurotransmitter levels) and external noise (e.g. eye blinks) lead to alternations between the two attractors. For model comparison, we chose the model presented by [5] as an example of such ”attractor” models.

Lastly, we considered an ”intermediate” model proposed by [6], which relates transitions in bistable perception to both adaptation of the dominant neuronal population and noise.

Methods

Oscillator Model

Theoretical Background

Oscillator models of bistable perception are based on mutual inhibition and adaptation [7, 4] and posit that two distinct neuronal populations represent the two distinct percepts compatible with a bistable stimulus. First, these models incorporate strong mutual inhibition between the two neuronal populations, which ensures that only one neuronal population will be active at any given time between perceptual transitions, thereby producing two mutually exclusive percepts. Second, these models incorporate adaptation of each neuronal population, meaning that the active neuronal population will eventually decrease in activity so that the suppressed neuronal population will become active, thereby producing perceptual transitions. Here, we implemented one established mutual-inhibition and adaptation model suggested by [4]. Crucial to our purpose, this model permits the estimation of the timepoints of perceptual transitions as a function of the underlying parameters, which allowed us to fit the model to behavioural data.

[4] assumes a simple threshold nonlinearity for the neural bistable perception model:

$$[X]_+ = max(X, 0) \tag{1}$$

The model (adapted for clockwise and counter-clockwise rotation of the Lissajous figure) is defined by four differential equations:

$$\tau \frac{dE_{Left}}{dt} = -E_{Left} + M[Left(t) - aE_{Right} + eE_{Left} - gH_{Left}]_{+} \quad (2)$$

$$\tau_H \frac{dH_{Left}}{dt} = -H_{Left} + E_{Left} \quad (3)$$

$$\tau \frac{dE_{Right}}{dt} = -E_{Right} + M[Right(t) - aE_{Left} + eE_{Right} - gH_{Right}]_{+} \quad (4)$$

$$\tau_H \frac{dH_{Right}}{dt} = -H_{Right} + E_{Right} \quad (5)$$

Equations 2 and 3 describe the activity of neurons responding to clockwise rotation. Here, E_{Left} describes the activity of neurons responsive to clockwise rotation, which are driven by the input $Left(t)$ and are inhibited by activity of neurons responsive to counter-clockwise rotation E_{Right} according to the inhibitory synaptic strength a ($-aE_{Right}$). Furthermore, E_{Left} is driven by a weak recurrent excitatory contribution defined by E_{Left} as well as a excitatory synaptic strength e ($+eE_{Left}$), and by self-adaptation defined by a slow self-depolarizing current H_{Left} and gain g ($-gH_{Left}$).

The time constant for the evolution of E_{Left} is given by τ (approx. 15 ms), whereas the time constant for the adaptation H_{Left} is described by τ_H (approx. 1000 ms). M describes the increase in neural firing rate as the excitatory input increases.

Equation 4 and 5 represent analogous differential equations for neurons responding to counter-clockwise rotation during ambiguous stimulation. The inputs $Left(t)$ and $Right(t)$ were set to 1 for ambiguous stimulation.

Under simplifying assumptions ($\tau << \tau_H$, [4]), it can be shown that:

$$E_{Left}(t) = \frac{M \times Left(t)}{1 - Me} - \frac{M^2 g \times Left(t)}{(1 - Me)(1 + Mg - Me)} \times (1 - exp(-\frac{(1 + Mg - Me)t}{(1 - Me)\tau_H})) \quad (6)$$

Here, t denotes the beginning of dominance period. Likewise, H_{Right} is given by:

$$H_{Right}(t) = \frac{M \times Right(t)}{1 + Mg - Me} \times exp(-t/\tau_H) \quad (7)$$

Analogous equations hold for E_{Right} and H_{Left}. With these expressions, the time of perceptual transitions can be calculated. This will occur when the $[X]+$ function for E_{Left} or E_{Right} just

reaches zero, respectively:

$$Right(t) - aE_{Left}(t) - gH_{Right}(t) = 0 \tag{8}$$

or

$$Left(t) - aE_{Right}(t) - gH_{Left}(t) = 0 \tag{9}$$

Starting with the percept indicated by the participant at the beginning of an ambiguous block, perceptual transitions were predicted using Equation 8 and 9. We then used the resulting hidden variables $E_{Left}(t)$ and $E_{Right}(t)$ to predict the subjects response θ, which is 0 for perception of clockwise and 1 for counter-clockwise rotation:

$$\theta = \begin{cases} 1: & \rightarrow \quad (rotation) \\ 0: & \leftarrow \quad (rotation) \end{cases} \tag{10}$$

In order to obtain a prediction of the subjects in the unit interval ($y_{predicted}$), we calculated calculated the sigmoid transform the the difference between $E_{Left}(t)$ and $E_{Right}(t)$ at every

timepoint:

$$y_{predicted}(t) = sigmoid(E_{Right}(t) - E_{Left}(t)) \tag{11}$$

$y_{predicted}(t)$ was then used to predict the perceptual outcome $\theta(t)$.

Since replay stimulation was not included in the original model [4], we added the following equation to account for disambiguated visual displays: In the disambiguated replay blocks, clockwise and counter-clockwise stimulation were defined as

$$simulation(t) = \begin{cases} 1: & \rightarrow \quad (stimulation) \\ 0: & \leftarrow \quad (stimulation) \end{cases} \tag{12}$$

$y_{predicted}$ for replay stimulation was then defined by applying the parameter d, accounting for the effectiveness of disambiguation:

$$y_{predicted} = \frac{exp(-\frac{d}{2} * (stimulation - 1)^2)}{exp(-\frac{d}{2} * (stimulation - 1)^2) + exp(-\frac{d}{2} * (stimulation)^2)} \tag{13}$$

Here, for $lim_{d\to\infty}$ (i.e. very effective disambiguation), $y_{predicted}(t)$ approaches 0 or 1, depending on $simulation(t)$. For $lim_{d\to 0}$ (i.e. less effective disambiguation), $y_{predicted}(t)$ approaches 0.5.

Model simulation

To establish that our implementation of the mutual inhibition and adaptation model [4] was able to reproduce the temporal dynamics of bistable perception, we simulated perceptual time-courses from some ambiguous input such as the Lissajous figure. We simulated for a total of $40 * 10^5$ ms and assumed $a = 3.4$, $g = 3$, $M = 1$, $\tau_H = 1000$. We calculated the distribution of dominance durations as well as the time-courses for E_{Left} and E_{Right}.

Modelling analysis of bahavioural data

In analogy to our modelling analysis using our predictive coding model, we fitted the mutual inhibition and adaptation model to the behavioural data collected during the fMRI experiment. Again, we optimized our model for the prediction of participants' responses that were aligned to the overlapping stimulus configurations of the Lissajous figure. For model inversion, parameters were modelled as log-normal distributions. a, g and d were estimated as free parameters (a: prior mean of log(3.4) and prior variance of 10; g: prior mean of log(3) and prior variance of 10; d: prior mean of log(10) and prior variance of 1). All other parameters were fixed to the values reported in [4] (see Supplementary Table 1). Parameters were optimised using quasi-Newton Broyden-Fletcher-Goldfarb-Shanno minimisation as implemented in the HGF4.0 toolbox.

To investigate whether the model implemented here adequately captured individual perceptual

dynamics during bistable perception, we analyzed the model's posterior parameters using classical frequentist statistics. Specifically, we calculated the Pearson correlation between conventional transition probabilities during ambiguous stimulation and a-posteriori a/g (the posterior inhibitory synaptic strength divided by the posterior hyperpolarizing current strength).

Attractor Model

Theoretical Background

Furthermore, we aimed at comparing our prediction error model to an attractor model of bistable perception, in which noise is crucial for the emergence of transitions in perception. In the model developed by [5], internal and external sources of noise cause switches between two stable states of the neuronal dynamics, which represent the two alternative and mutually exclusive percepts associated with bistable perception. According to this notion, perception would hence cease to fluctuate in the absence of noise, despite the ambiguity of the sensory signal.

[5] posit that the dominant states of the two populations coding for the alternative percepts A_{on} and B_{on} are defined by their mean firing rates r_A and r_B: $A(r_A >> r_B)$ and $B(r_B >> r_A)$. The attractor states are then described by an energy function with two minima, representing A_{on} and B_{on}:

$$E(\Delta r) = \Delta r^2(r^2 - 2) + g_a(\Delta r + 1)^2 + g_B(\Delta r + 1)^2 \quad (14)$$

Here, Δr denotes the difference in the mean firing rate ($r_A - r_B$), while g_a and g_B denote the input strengths.

The dynamic variable r moves along the horizontal axis of this energy function. Since its first derivative is zero at the minima, those points represent the stable attractors. The temporal evolution of Δr is given by:

$$\tau \frac{d}{dt}(\Delta r) = -4\Delta r(r^2 - 1) + g_a(\Delta r - 1) + g_B(\Delta r + 1) + n(t) \tag{15}$$

τ represents the timescale of changes in Δr. The noise term $n(t)$ is a Ornstein-Uhlenbeck process with zero mean and deviation σ.

$$\frac{d}{dt}n = -\frac{n}{\tau_s} + \sigma\sqrt{(\frac{2}{\tau_s})}\zeta(t) \tag{16}$$

Importantly, $\zeta(t)$ represents a white noise process with zero mean and variance v. Please note that in the absence of noise ($v = 0$), the system settles in one of the two attractors.

We used Δr to predict the two perceptual outcomes at each overlapping configuration of the Lissajous figure:

$$\theta = \begin{cases} 1: & \rightarrow \quad (rotation) \\ 0: & \leftarrow \quad (rotation) \end{cases} \tag{17}$$

To this end, z-transformed Δr was thresholded to satisfy the relations $A(r_A >> r_B)$ and $B(r_B >> r_A)$:

$$y_{predicted}(t) = \begin{cases} 1: & \Delta r > threshold \\ 0: & \Delta r < -threshold \end{cases} \tag{18}$$

In analogy to our implementation of the mutual-inhibition and adaptation model [4], responses during replay were predicted by applying parameter d as a measure of the effectiveness of disambiguation:

$$simulation(t) = \begin{cases} 1: & \rightarrow \quad (stimulation) \\ 0: & \leftarrow \quad (stimulation) \end{cases} \tag{19}$$

$$y_{predicted} = \frac{exp(-\frac{d}{2} * (stimulation - 1)^2)}{exp(-\frac{d}{2} * (stimulation - 1)^2) + exp(-\frac{d}{2} * (stimulation)^2)} \tag{20}$$

Model simulation

To make sure that our implementation of the noise-driven attractor model [5] was able to reproduce the temporal dynamics of bistable perception, we simulated perceptual perceptual decisions for a total of a total of $40 * 10^5$ ms and assumed $\tau = 10$, $g_B = g_A = 1$, $v = 0.0001$, $\tau_s = 50$, $\sigma = 0.7$ and $threshold = 0.75$. We calculated the distribution of dominance durations as well as the time-course of Δr.

Modelling analysis of behavioural data

Modeling analysis was carried out in analogy to the mutual-inhibition and adaptation model ([4], see above). For model inversion, parameters were modelled as log-normal distributions. v and d were estimated as free parameters (v: prior mean of log(0.1) and prior variance of 1; d: prior mean of log(10) and prior variance of 1). All other parameters were fixed (see Supplementary Table 2). Parameters were optimised using quasi-Newton Broyden-Fletcher-Goldfarb-Shanno minimisation as implemented in the HGF4.0 toolbox.

To investigate whether the model implemented here adequately captured individual perceptual dynamics during bistable perception, we analyzed the model's posterior parameters using classical frequentist statistics. Specifically, we calculated the Pearson correlation between conventional transition probabilities during ambiguous stimulation and a-posteriori v (the variance of the white Gaussian noise representing the driving force between perceptual transitions during bistable perception according to [5]).

Intermediate Model

Theoretical Background

Finally, we sought to compare our model to an intermediate model that incorporates both adaptation and noise. To this end, we chose the model put forward by [6]. This model was designed to match the structure of electronic astable mulitvibrators and produces transitions between two perceptual states associated with a bistable stimulus.

In this framework, the mean activity of the neuronal population coding for the currently dominant percept evolves according to:

$$dominant_{t+\Delta t} = a * dominant_t + b * \delta \tag{21}$$

Here, δ is set to +1.0 or -1.0 with probability 0.5 at each timestep. a denotes the strength of

adaptation and b scales the amount of noise desired in the system.

Conversely, the mean activity of the neuronal population coding for the currently suppressed percept is given by:

$$suppressed_{t+\Delta t} = a * suppressed_t + (1 - a) + b * \delta \tag{22}$$

Once the activity of the dominant population reaches a threshold ($dominant_t = threshold$), the system reverses.

We extracted the modelled activity of the neuronal population coding for counter-clockwise rotation $activity_{\theta=1}$. For replay stimulation, $activity_{\theta=1}$ was set to 1 for counter-clockwise stimulation and to 0 for clockwise stimulation. The perceptual outcomes were predicted by applying a sigmoid transform to z-normalized $activity_{\theta=1}$.

$$y_{predicted}(t) = sigmoid(zscore(activity_{\theta=1}(t))) \tag{23}$$

Model simulation

To verify that our implementation of the intermediate model [6] was able to reproduce the temporal dynamics of bistable perception, we simulated perceptual perceptual decisions for a total of a total of $40 * 10^5$ ms and assumed $a = 0.9048$, $b = 0.1$ and $threshold = 0.2$. We

calculated the distribution of dominance durations as well as the time-course of mean activity in the neuronal population coding for counter-clockwise rotation.

Modelling analysis of behavioural data

Modeling analysis was carried out in analogy to the mutual-inhibition and adaptation model ([4], see above). For model inversion, parameters were modelled as log-normal distributions. a and b were estimated as free parameters (a: prior mean of log(0.9048) and prior variance of 0.1; b: prior mean of log(0.1) and prior variance of 0.1). The parameter $threshold$ was kept fixed at 0.2. Parameters were optimised using quasi-Newton Broyden-Fletcher-Goldfarb-Shanno minimisation as implemented in the HGF4.0 toolbox.

To investigate whether the model implemented here adequately captured individual perceptual dynamics during bistable perception, we analyzed the model's posterior parameters using classical frequentist statistics. Specifically, we calculated the Pearson correlation between conventional transition probabilities during ambiguous stimulation and a-posteriori $a * b$ (the strength of adaptation times the amount of noise within in the system [6]).

Bayesian Model Comparison

Most importantly, in a final step, we aimed to compare the performance of our predictive coding model to the performance of the established models described above. We therefore performed Random Effects Bayesian Model Comparison [8], as implemented in SPM12 (http://www.fil.ion.ucl.ac.uk/spm/software/spm12) between predictive coding, oscillator, attractor and interme-

diate models, reporting expected ratios and protected exceedance probabilities.

Results

We used data simulations in order to establish whether our implementation of the oscillator, attractor and intermediate models were able to reproduce perceptual time-courses similar to bistable perception. Indeed, the distribution of perceptual phase durations followed a sharp rise and slow fall for all models (Figure 1 a-c), which is typical for bistable perception [9, 10]. Mean and median simulated phase durations amounted to 1086 and 1084 ms for the oscillator model, 2413 and 1814 ms for the attractor model as well as 1486 and 1300 ms for the intermediate model. The insets in Figure 1A and C show simulated time-courses of neuronal activity in neurons coding for clockwise and counter-clockwise rotation. The inlet in Figure 1B shows an exemplary time-course for Δ_r.

To investigate whether the models described above were able to explain the dynamics of perceptual bistability in human observers, we tested whether the a-posterior model parameters correlated with conventional transition probabilities during ambiguous sitmulation.

For the oscillator model, a/g (the posterior inhibitory synaptic strength divided by the posterior hyperpolarizing current strength) was positively correlated with perceptual transition frequencies across participants ($\rho = 0.5750$, $p < 0.0080$, Pearson correlation, Fig.1D), suggesting that the model fitted the behavioral data. Furthermore, this correlation was also significant when we correlated model parameter estimates for a/g averaged over run 1 and 2 with perceptual transition frequencies from run 3 (Pearson correlation to a/g: $\rho = 0.6314$, $p < 0.0028$, Fig.

1G), indicating that the mutual-inhibition and adaptation model also accounted for observers' perception evoked by an ambiguous stimulus. Overall, a-posteriori a amounted to 12.6076 ± 2.4048 ms (standard error of the mean) across observers. A posteriori g was 9.0257 ± 1.9121 on average. A-posteriori d amounted to 2.6064 ± 0.3027 ms across observers.

For the noise-driven attractor model, the a-posteriori value of v (variance of the Gaussian noise) did not correlate with conventional transition probabilities (see Figure 1E and H), indicating that this model was unable to capture the perceptual dynamics in this specific experiment using the Lissajous Figure. In this model, a-posteriori value v amounted to 0.1582 ± 0.0223 (standard error of the mean) across observers. A-posteriori d amounted to 1.1569 ± 0.3512 across observers.

For the intermediate model, we correlated the product from a-posteriori a (strength of adaptation) and a-posteriori b (the amount of noise within in the system) with conventional transition probabilities. Here, we found a significant positive Pearson correlation ($\rho = 0.5355$, $p < 0.0140$, Fig. 1F) between the two measures. Additionally, this correlation was also borderline-significant when we correlated model parameter estimates for $a * b$ averaged over run 1 and 2 with transition probabilities from run 3 (Pearson correlation to $a * b$: $\rho = 0.4381$, $p < 0.0533$, see Fig. 1I), indicating that the intermediate model successfully accounted for individual perceptual time-courses during ambiguous stimulation.

In a final step, we compared our predictive coding model to the oscillator, attractor and intermediate model using Random Effects Bayesian Model Comparison. This analysis identified our predictive coding model as a winning model with an expected ratio of 0.8303 and an protected

exceedance probability of 99.99% (Fig. 2A and B).

Discussion

In this supplementary analysis, we compare our predictive coding approach to three established models of bistable perception based on adaptation and inhibition [4], noise [5] and an intermediate model [6]. While all three models were able to reproduce the known characteristics of bistable perception, only the adaptation and inhibition as well as the intermediate model successfully predicted participants perception during viewing of ambiguous and unambiguous visual Lissajous stimuli. Conversely, a-posteriori parameters from the noise-driven attractor model did not correlate with conventional measures of transition frequency.

One possibility for this lack of model-fit in the noise-driven attractor model might the regularity of perceptual transitions in the Lissajous figure, which almost uniquely occur at overlapping configurations of the stimulus [11]. Models that rely only on noise as the driving force behind perceptual transitions [5] and do not incorporate some sort of temporal regularity such as adaptive processes [6, 4] thus might be unable to explain the perceptual dynamics of this particular stimulus.

Crucially, formal model comparison between the established models and our predictive coding model revealed a superiority of the latter, given the data collected in this study. Importantly, this model comparison result does not indicate that our modelling approach is superior to oscillator, attractor or intermediate models. This is because the models described here were developed to explain neuronal time-courses during bistable perception and were not designed to predict per-

ceptual decisions during bistable perception timepoint-by-timepoint. Furthermore, the original models did not include a replay condition, which we added in order to enable a full model comparison. Finally, the the earlier models were designed to explain perceptual time-courses mostly during binocular rivalry or continuous structure-from-motion, which are different to our stimulus in various respects, most importantly due to increased transition probabilities at overlapping stimulus configurations of the Lissajous figure (see above).

Taken together, the results of our supplementary analyses suggest that - given the specific behavioural data collected in this experiment - our predictive coding model is at least equivalent to established models of bistable perception in explaining perceptual decision during viewing of an ambiguous stimulus.

Figures

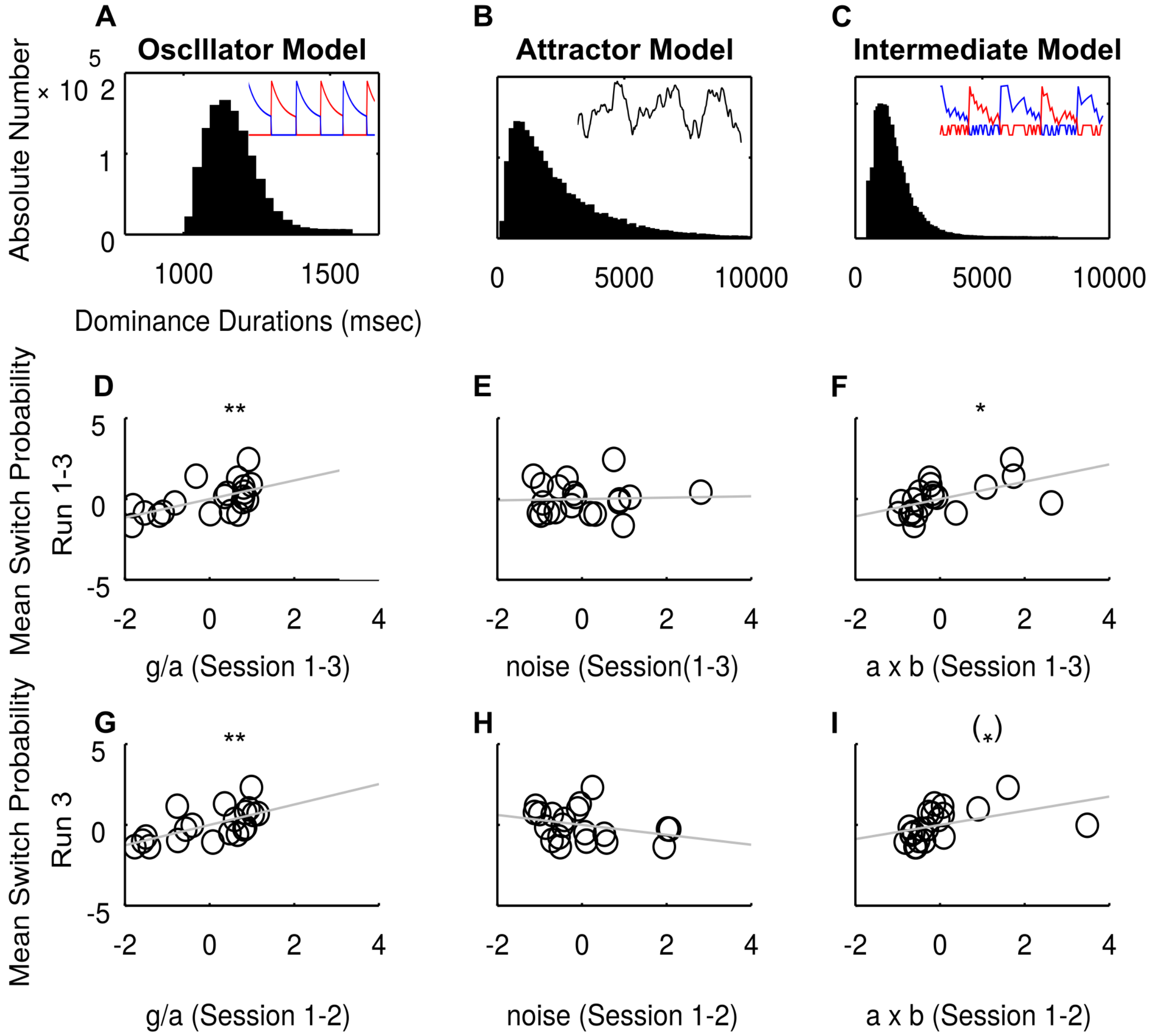

Supplementary Figure S1. A-C: Simulating perceptual decisions during ambiguous stimulation. Data were simulated for a total of $40 * 10^5$ ms. **A:** Oscillator model with $a = 3.4$, $g = 3$, $M = 1$, $\tau_H = 1000$. The distribution of phase durations was characterized a sharp rise and slow fall resembling a gamma-distribution with a mean dominance duration of 1086 ms. The inlet shows simulated time-

courses for neurons coding for counter-clockwise (blue) and clockwise (red) rotation. **B:** Noise-driven attractor model with $\tau = 10$, $g_B = g_A = 1$, $v = 0.0001$, $\tau_s = 50$, $\sigma = 0.7$ and $threshold = 0.75$. The distribution of phase durations was characterized a sharp rise and slow fall resembling a gamma-distribution with a mean dominance duration of 2413 ms. The inlet shows the simulated time-course for the rate difference Δ_r in black. **C:** Intermediate model with $a = 0.9048$, $b = 0.1$ and $threshold = 0.2$. The distribution of phase durations was characterized a sharp rise and slow fall resembling a gamma-distribution with a mean dominance duration of 1486 ms. The inlet shows the simulated time-course for neurons coding for counter-clockwise (blue) and clockwise (red) rotation. **D-I: Correlation between posterior parameters of participants' behaviour. D**: Transition probabilities form the oscillator model correlated significantly with posterior a/g across participants ($\rho = 0.5750$, $p < 0.0080$), providing a sanity check for model fit. **E**: Transition probabilities did not correlate significantly with posterior v from the noise-driven attractor model. **F**: Transition probabilities form the intermediate model correlated significantly with posterior $a * b$ across participants ($\rho = 0.5355$, $p < 0.0140$,), providing a sanity check for model fit. **G**: Transition probabilities from run 3 were predictive of posterior a/g from the oscillator model averaged over run 1 and 2 ($\rho = 0.6314$, $p < 0.0028$), illustrating the explanatory power of this model. **H**: Transition probabilities from run 3 did not correlate significantly with posterior v from the noise-driven attractor model averaged over run 1 and 2. **I**: Transition probabilities from run 3 were predictive of posterior $a * g$ from the intermediate model averaged over run 1 and 2 ($\rho = 0.4381$, $p < 0.0533$), illustrating the explanatory power of this model.

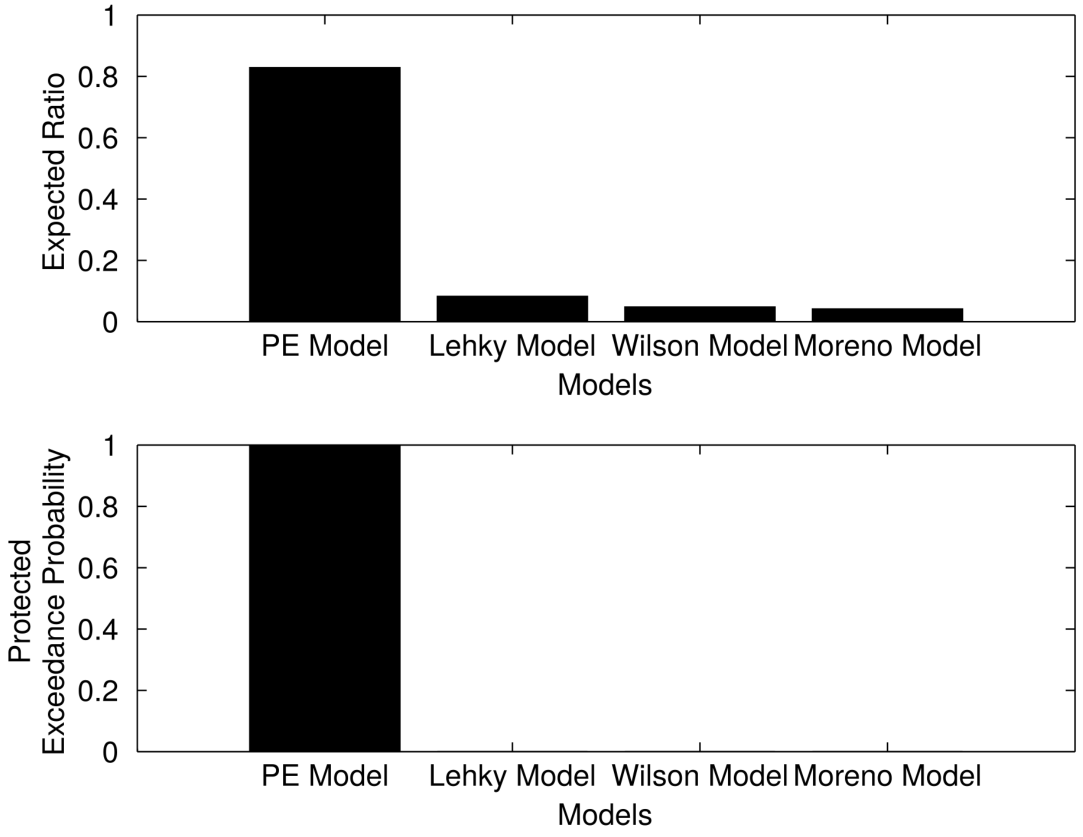

Supplementary Figure S2. Random Effects Bayesian Model Comparison of our predictive coding model to oscillator, attractor and intermediate model. The predictive coding model was identified as superior (**A**: expected ratio; **B**: protected exceedance probability) in explaining participants' behaviour.

Supplementary Table 1: Parameters of the mutual inhibition and adaptation model

**Parameter**	**Explanation**	**Prior Mean**	**Prior Variance**
τ	Excitatory neural time constant	15 ms	0
τ_H	Hyperpolarizing time constant	1000 ms	0
M	Response gain constant	1	0
a	Inhibitory synaptic strength	3.4	10
g	Hyperpolarizing current strength	3.0	10
e	Excitatory synaptic strength	0	0
Left(t)	Time course of clockwise input	1	0
Right(t)	Time course of counter-clockwise input	1	0
d	Effectivity of perceptual disambiguation	10	1

Supplementary Table 2: Parameters of the noise-driven attractor model

Parameter	Explanation	Prior Mean	Prior Variance
τ	Δr time constant	10 ms	0
τ_s	Noise time constant	50 ms	0
v	Noise variance	0.1	1
g_A	Input strength percept A	1	0
g_B	Input strength percept B	1	0
threshold	Threshold for predicted responses	0.75	0
d	Effectivity of perceptual disambiguation	10	1

References

1. Stollenwerk L, Bode M. Lateral Neural Model of Binocular Rivalry. Neural Computation. 2003;15(12):2863–2882. doi:10.1162/089976603322518777.

2. Matsuoka K. The dynamic model of binocular rivalry. Biological cybernetics. 1984;49(3):201–8.

3. Laing CR, Chow CC. A spiking neuron model for binocular rivalry. Journal of computational neuroscience;12(1):39–53.

4. Wilson HR. Minimal physiological conditions for binocular rivalry and rivalry memory. Vision research. 2007;47(21):2741–50. doi:10.1016/j.visres.2007.07.007.

5. Moreno-Bote R, Rinzel J, Rubin N. Noise-Induced Alternations in an Attractor Network Model of Perceptual Bistability. Journal of Neurophysiology. 2007;98(3):1125–1139. doi:10.1152/jn.00116.2007.

6. Lehky SR. An astable multivibrator model of binocular rivalry. Perception. 1988;17(2):215–28.

7. Blake R. A neural theory of binocular rivalry. Psychological review. 1989;96(1):145–67.

8. Stephan KE, Penny WD, Daunizeau J, Moran RJ, Friston KJ. Bayesian model selection for group studies. NeuroImage. 2009;46(4):1004–17. doi:10.1016/j.neuroimage.2009.03.025.

9. Levelt WJM. Note on the Distribution of Dominance Times in Binocular Rivalry. British Journal of Psychology. 1967;58(1-2):143–145. doi:10.1111/j.2044-8295.1967.tb01068.x.

10. Logothetis NK, Leopold DA, Sheinberg DL. What is rivalling during binocular rivalry? Nature. 1996;380(6575):621–4. doi:10.1038/380621a0.

11. Weilnhammer VA, Ludwig K, Sterzer P, Hesselmann G. Revisiting the Lissajous figure as a tool to study bistable perception. Vision research. 2014;98:107–12. doi:10.1016/j.visres.2014.03.013.
